# The role of SIGLEC9 in immunosuppression and prognosis in cervical cancer

**DOI:** 10.1016/j.clinsp.2025.100849

**Published:** 2025-12-18

**Authors:** Bihui Wang, Yuejie Zhu, Zhenyu Ru, Yulian Zhang, Mingkai Yu, Pingfen Li, Manli Zhang, Jianbing Ding, Zhifang Chen

**Affiliations:** a Department of Gynecology, the First Affiliated Hospital of Xinjiang Medical University, Urumqi, China; b State Key Laboratory of Pathogenesis, Prevention and Treatment of High Incidence Diseases in Central Asia, the First Affiliated Hospital of Xinjiang Medical University, Urumqi, China; c Center for Reproductive Medicine, First Affiliated Hospital of Xinjiang Medical University, Urumqi, China; d Department of Gastroenterology, the First Affiliated Hospital of Xinjiang Medical University, Urumqi, China; e School of Life Science and Technology, Southeast University, Nanjing, China; f Department of Reproductive Medicine, The First Affiliated Hospital of Xi'an Jiao Tong University, Shanxi, Xi’an, China; g Department of Immunology, College of Basic Medicine of Xinjiang Medical University, Urumqi, China

**Keywords:** Cervical Cancer (CC), Sialic Acid-Binding Immunoglobulin-Like Lectin-9 (SIGLEC9), Mucin 1 (MUC1), T-cells, Tumor-Associated Macrophages (TAMs)

## Abstract

**Objective:**

This study investigates the role of Sialic acid-binding Immunoglobulin-Like Lectin-9 (SIGLEC9), a novel immune checkpoint, in Cervical Cancer (CC) and its interaction with immune cells in the tumor microenvironment.

**Methods:**

SIGLEC9 expression in CC was analyzed using the TNM plot, TCGA, and Human Protein Atlas databases, alongside its correlation with tumor stage. The relationship between SIGLEC9 expression and immune cell infiltration was explored using TCGA and TISIDB databases. Single-cell analysis focused on SIGLEC9 in macrophages. Protein interactions were assessed through the String, IntAct, BioGRID, and Mentha databases. MUC1 expression was validated via GEO and GEPIA databases. Immunohistochemical staining, western blot, immunofluorescence, and flow cytometry were used for verification. The prognostic significance of SIGLEC9 and SIGLEC9+ Tumor-Associated Macrophages (TAMs) was evaluated.

**Results:**

SIGLEC9 was found to be significantly upregulated in CC, with higher levels correlating with poor prognosis. It was expressed in macrophages and T-cells, and elevated SIGLEC9+ TAMs were linked to reduced overall survival.

**Conclusion:**

SIGLEC9 plays a crucial role in the progression and prognosis of cervical cancer through its interaction with TAMs and T-cells. These findings highlight SIGLEC9 as a potential target for new immunotherapies in CC.

## Highlights

• SIGLEC9 is highly expressed in cervical cancer and linked to poor prognosis.
• Bioinformatics show SIGLEC9 correlates with macrophages and T-cells.
• SIGLEC9+ TAMs and T-cells are highly enriched in the cervical cancer microenvironment.
• High SIGLEC9+ TAM expression correlates with poor prognosis in cervical cancer.

## Introduction

Despite advances in screening and treatment of cervical cancer, it remains the fourth most common disease and the leading cause of cancer deaths in women worldwide.^1^ Adaptive Cell Therapy (ACT) and Immune Checkpoint Inhibitors (ICIs) (such as PD-1/PD-L1) have dramatically prolonged the survival of cervical cancer patients.^2^ Although anti-PD-1/PD-L1 monoclonal antibodies have achieved outstanding clinical results in the treatment of specific cancer types, their overall response rate is still rather mediocre (12.46 % in US patients in 2018).^3^ Therefore, the authors need to translate more drug candidates into the clinical arena through more ways and methods to achieve greater success and benefit more patients.

*Sci Transl Med* reports that the Sialoglycan/Sialic acid binding Immunoglobulin-like Lectins (SIGLECS) axis is a novel immune checkpoint whose changes greatly influence the stability of the immune system, and that targeting this axis can drive both innate and adaptive anti-tumor immunity in the body.^4^^,^^5^ The CD33-related Silane-Acid-Binding Immunoglobulin-like Lectin 9 (SIGLEC9) is of particular interest in tumor immunotherapy,^6^ which showed high affinity for α−2,3 and α−2,6 binding sialoglycans.^7^ Emerging evidence suggests that SIGLEC9 could be considered as a new immune checkpoint and target for cancer immunotherapy.^8^ It is expressed on NK cells, T-cells, tumor-associated macrophages, neutrophils, and MDSC.^8^^,^^9^ Among them, SIGLEC-9 was observed preferentially on myeloid cells.^10^ SIGLEC9 has been identified as an immune checkpoint molecule on Tumor-Associated Macrophages (TAMs).^11^ Tumor-derived sialic acids dictate monocyte to macrophage differentiation via signaling through SIGLEC9, and provide new potential targets for cancer immunotherapy in PDAC.^12^ Moreover, MUC1-ST induced macrophages to display a Tumor-Associated Macrophage (TAM)-like phenotype, with increased expression of the checkpoint ligand PD-L1.^13^ In addition, SIGLEC9 is also associated with T-cells. Quentin Haas demonstrated that the majority of tumor-infiltrating T-cells in melanoma specimens expressed SIGLEC9. It acts as a suppressor of T-cell effector responses in the tumor microenvironment.^14^ Although the functions of SIGLEC9 have been explored in certain types of cancer, there is currently no research on its impact on tumor immunity in cervical cancer. Additionally, its potential as a prognostic biomarker for prediction, as well as its role as an adjuvant therapy to enhance Immune Checkpoint Blockade (ICB) response, has yet to be investigated.

Therefore, this study aims to clarify the expression and prognostic significance of SIGLEC9 in cervical cancer, while also exploring its correlation with Tumor-Associated Macrophages (TAMs) and T-cells in the tumor microenvironment. By addressing these gaps, the authors hope to identify potential therapeutic targets that could enhance the efficacy of immunotherapy for patients with cervical cancer.

## Materials and methods

### Data collection

The online analysis tool TNMplot database (https://tnmplot.com/analysis/) is utilized for comparing gene expression levels in normal, tumor, and metastatic tissues. With a repository of 57,000 samples, the TNMplot database includes an array of RNA-Seq and microarray datasets, making it the most extensive transcriptomic cancer database accessible. To examine the variances in mRNA expression of SIGLEC9 among normal tissues, tumors, and metastatic tissues, TNMPlot was employed in this investigation.

The Cervical Squamous Cell Carcinoma and Endocervical Adenocarcinoma (CESC) dataset containing gene expression profiles and clinical information was downloaded from the publicly available The Cancer Genome Atlas (TCGA) (https://portal.gdc.cancer.gov), and included 3 normal and 306 tumor tissues. Subsequent processing excluded cases with insufficient or missing data on age and overall survival time. The RNA sequencing data were then first transformed to convert the count data to values more similar to the microarray data. CESC patients were categorized into a low SIGLEC9 expression group and a high SIGLEC9 expression group based on the median SIGLEC9 expression value.

GSE9750, GSE26511 were downloaded from the Gene Expression Omnibus (GEO database) (https://www.ncbi.nlm.nih.gov/geo/). In this study, the authors analyzed the differences in MUC1 mRNA expression between normal and tumor tissues using these databases.

Gene Expression Profiling Interactive Analysis (GEPIA) (http://gepia.cancer-pku.cn/index.html), an online database that analyzes 306 tumors from the RNA sequencing expression data and 13 normal samples from projects known as the Genotype-Tissue Expression (GTEx) and TCGA. Furthermore, boxplots using disease state (Tumor or Normal) as a variable were graphed to calculate differential expression of MUC1.

### Patients and samples

A total of 58 patients with cervical cancer who underwent surgical treatment in the Department of Gynecology of the First Affiliated Hospital of Xinjiang Medical University were included in this study. All cervical cancer patients were diagnosed with cervical cancer for the first time and had not received any chemotherapy or other treatment before. Exclusion criteria: patients with diabetes mellitus, hypertension, cardiovascular disease, pregnancy and history of acute and chronic infectious diseases or metastatic tumors were excluded, as well as patients with cervical cancer who were undergoing radiotherapy before surgery. The study was approved by the Ethics Committee of the First Affiliated Hospital of Xinjiang Medical University (Ethics n° 220,525–02), and all subjects signed an informed consent form. The control group consisted of cervical tissues from 30 patients who underwent myomectomy during the same period and had negative cervical cancer screening. Patients with cervical cancer were staged according to the 2018 American Joint Committee on Cancer TNM staging system (AJCC). Tissue samples were frozen in liquid nitrogen after surgery and stored until analysis at −80 °C. Fresh tumor tissue samples obtained during gynecological surgery at the hospital in 40 patients with cervical cancer were used for Flow Cytometry (FCM), and 6 of the cervical cancer tissues were used for immunofluorescence. Clinical Trials should follow the CONSORT Statement rules. Diagnostic and Prognostic Studies should follow the STARD guidelines.

### Tumor immune analysis

To explore whether SIGLEC9 expression is associated with the tumor immune microenvironment, the authors first explored the abundance of immune and stromal cells between different groups using the ESTIMATE algorithm and calculated the StromalScore, ImmuneScore, and ESTIMATEScore (StromalScore + ImmuneScore). Next, the CIBERSORT algorithm was used to assess the level of invasion of the 22 immune cells in the samples and to evaluate the difference in the level of invasion of each immune cell between the SIGLEC9 high- and low-expression groups. CIBERSORT calculates the putative proportions of immune cells based on the gene expression profiles.^15^ The results obtained by CIBERSORT were screened with a screening value of *p* = 0.05.

### TISIDB

The TISIDB database (http://*cis*.hku.hk/TISIDB) is an open source website exploring the relationship between genes and tumor immunity interactions, which was completed by integrating >4176 records from 2530 publications to report 988 genes associated with antitumor immunity.^16^ The authors estimated the mRNA expression levels of SIGLEC9 in different immune subtypes using the TISIDB database and also analyzed the expression of the SIGLEC9 gene in different immune subtypes, which included C1 (wound healing), C2 (IFN-γ dominant), C3 (inflammation), C4 (lymphocyte depletion), C5 (immune-quieting) and C6 (TGF-β dominant) subtypes.

### Single-cell RNA sequencing analysis

GSE168652 includes the scRNA-seq data from 14,220 cells of CESC and 11,422 cells of the adjacent normal tissues. The scRNA-seq data underwent quality control and analysis using the Seurat (4.1.1) *R* package. Cells with fewer than 200 expressed genes, genes expressed in <3 cells, and over 10 % of mitochondrion-derived genes were filtered out. A set of 2000 variable genes was selected for Principal Components Analysis (PCA). The first 10 principal components were visualized using t-Distributed Stochastic Neighbor Embedding (t-SNE) plots after dimensionality reduction. Prior to clustering, the ScaleData function was applied to account for the influence of mitochondrion-derived genes. All cells were divided into 13 clusters with a resolution = 0.5, and further divided into seven subgroups based on published markers: cancer cells, endothelial cells, endometrial stromal cells, fibroblasts, lymphocytes, macrophages, and smooth muscle cells.^17^

### PPI network analysis

To further explore the functional relevance of SIGLEC9 in cancer, the authors compiled a list of genes that directly interact with SIGLEC9 from four protein-interaction network databases: STRING (http://www.string-db.org),^18^ BioGRID (https://thebiogrid.org), Mentha (https://mentha.uniroma2.it), and IntAct (https://ngdc.cncb.ac.cn/databasecommons/database/id/534).

### Functional enrichment analysis

Gene Ontology analysis (GO) encompasses three main domains: Biological Processes (BP), Cellular Components (CC), and Molecular Functions (MF). Utilizing the GO database, the biological functions of the target genes were identified for CC, MF, and BP. The Kyoto Encyclopedia of Genes and Genomes (KEGG), a comprehensive database that integrates genomic, chemical, and system functional information, was employed for biological pathway analysis. Both GO and KEGG analyses were conducted using the 'Hs.eg.db', 'clusterProfiler', and 'enrichplot' packages. Statistical significance was determined with a threshold of *p* < 0.05 and FDR < 0.05 for enriched biological processes and pathways.

### Function and pathway analysis by Gene Set Enrichment Analysis (GSEA)

This study aimed to explore the role of SIGLEC9 in CESC through Gene Set Enrichment Analysis (GSEA). Gene Set Enrichment Analysis (GSEA) is a valuable method for elucidating the underlying biological mechanisms of genes. Differentially Expressed Genes (DEGs) between groups with low and high SIGLEC9 expression levels were identified using the DESeq2 *R* package (version 1.26.0). In this study, GSEA was conducted utilizing the ggplot2 *R* package (v3.3.3) to illustrate the significant functions and pathways between these two groups. The expression level of SIGLEC9 served as the phenotype label. Criteria for significance included an adjusted p-value < 0.05, a Normalized Enrichment Score (|NES|) > 1, and a False Discovery Rate (FDR) < 0.25.

### HPA analysis

The protein expression of SIGLEC9 was obtained from the Human Protein Atlas (HPA) (http://www.proteinatlas.org/) database.

### Cell culture

Immortalized human cervical epithelial cells (H8 cells), human immortalized epidermal Cells (HacaT cells), and cervical cancer cell lines HeLa and SiHa cells were purchased from the Cell Bank of the Chinese Academy of Science (Shanghai, China). H8, HacaT, HeLa, and SiHa cells were cultured in high-glucose DMEM (Bioscience, China) supplemented with 10 % Fetal Bovine Serum (FBS; EVERY GREEN, China) and 1 % penicillin-streptomycin (Bioscience, China) under standard conditions at 37 °C in a 5 % CO_2_ humidified atmosphere.

### Immunohistochemical staining (IHC)

The authors examined the protein level expression of SIGLEC9 and MUC1 by IHC. All participants provided informed consent prior to the study. Tumor samples, specifically surgically resected cervical cancer tissues, were preserved in Formalin-Fixed-Paraffin-Embedded (FFPE) blocks. FFPE slides were baked for 4 h, deparaffinized with dimethylbenzene, and dehydrated through an ethanol gradient. Antigen retrieval was performed using boiled Tris-EDTA (pH 9.0, Solarbio, China), followed by blocking of cyclooxygenase with 3 % hydrogen peroxide and nonspecific antigens with 10 % goat serum (ZSGB, China). The primary antibodies were diluted as follows: rabbit anti-human SIGLEC9 (1:200, Proteintech, China) and MUC1 (1:200, Boster, China) and incubated overnight at 4 °C. On the following day, slides were incubated with horseradish peroxidase-labeled secondary antibodies (1:200, Proteintech, China) at 37 °C for 1 h. The slides were stained with DAB (ZSGB, China) working solution (prepared and stored in the dark), and the nuclei were counterstained with hematoxylin (Solarbio, China) for 2 min, then rinsed in running water to return to blue. The slides were counterstained, dehydrated, and coverslipped. Protein expression was observed and photographed under a microscope (Nikon, Japan). The FIJI version 2.9.0 software was utilized for image analysis, including optical density correction, selection of positively stained areas in HIS color mode, filtering of background interference, measurement of Integral Optical Density (IOD) values, and positive areas. Five complete and non-overlapping high-power fields of view (× 200) were randomly selected from each slide, and the IOD and area values of the positive area were measured under each field of view. The average optical density (MOD) was calculated as MOD = IOD/Area. The average MOD values of all fields of view within each group were used as the group measurement values.

### Western blotting

Total protein was extracted from human cervical tissues using RIPA buffer (Solarbio, China) containing a commercial protease inhibitor (Solarbio, China) and a phosphatase inhibitor (Proteintech, China) in a 1:1:100 ratio. Protein concentration was determined using the BCA protein quantification assay (Solarbio, China). The protein samples were then heated at 100 °C for 10 min. SDS-PAGE electrophoresis was performed at 10 % concentration for 2 h, followed by membrane transfer onto PVDF membranes (Millipore, Germany) for 1 h. The membranes were incubated with 5 % skim milk for 1 h. Subsequently, membranes were incubated overnight at 4 °C with primary antibodies: rabbit anti-human SIGLEC9 (1:1000, Proteintech, Chinav) and GAPDH (1:3000, Proteintech, China). On the following day, the membranes were treated with horseradish peroxidase-conjugated secondary antibodies (1:5000, Proteintech, China) for 1 h. Signal detection was performed using an enhanced chemiluminescence detection system (Biosharp, China) according to the manufacturer’s instructions. Signal intensity was analyzed using densitometric scanning (Azure 280–600, USA), and FIJI version 2.9.0 software was used to quantify the gray values of the target proteins relative to the reference protein (GAPDH). The ratio of the target protein gray values to the GAPDH gray values reflected the expression level of the target protein.

### Human double immunofluorescence

FFPE slides were baked for 4 h, deparaffinized with dimethylbenzene, and dehydrated with a gradient of ethanol. The sections were then placed in Tris-EDTA antigen repair solution (pH 9.0, Solarbio, China) and subjected to microwave heating at 95 °C for 15 min to facilitate antigen retrieval. Following natural cooling to room temperature, the sections were washed three times with phosphate buffer for 5 min each. Goat serum (Zhongshan Jinqiao, China) was used for blocking, followed by incubation at room temperature for 60 min. After the serum was removed, anti-CD4 (1:200, Ptroteintech, China) and SIGLEC9 (1:200, Proteintech, China) antibodies were added dropwise to the paraffin sections. The sections were then incubated at 4 °C overnight, followed by a 1 h incubation at room temperature the next day. Subsequently, CoraLite594-conjugated Goat Anti-Mouse IgG (*H* + *L*) (1:200, Proteintech, China) and CoraLite488-conjugated Goat Anti-Rabbit IgG (*H* + *L*) (1:200, Proteintech, China) fluorescent secondary antibodies were added, and the sections were incubated at 37 °C in the dark for 30 min. After incubation, DAPI (Solarbio, China) was added dropwise, and the sections were incubated in the dark at room temperature for 10 min. Between these steps, the sections were rinsed three times with TBST for 5 min each. Finally, images were captured using a Nikon confocal AX nsparc microscope (Nikon, Japan) after the antiquenching agent was added to the tablet. The mean gray values (mean) of CD4 (red) and SIGLEC9 (green) were analyzed using a color image analysis system (FIJI 2.9.0) and calculated as Mean = integrated density (IntDen) / area. The relative fluorescence intensity of the two groups was calculated using Version 9.3.1 software (GraphPad Software) (relative fluorescence intensity of cervical cancer group = mean of cervical cancer group/average mean of control group; relative fluorescence intensity of normal control group = mean of normal control group/average mean of control group).

FFPE slides were baked for 4 h, deparaffinized with dimethylbenzene, and dehydrated using an ethanol gradient. The sections were then placed in Tris-EDTA antigen repair solution (pH 9.0, Solarbio, China) and subjected to microwave heating at 95 °C for 15 min to facilitate antigen retrieval. Following natural cooling to room temperature, the sections were washed three times with phosphate buffer for 5 min each. Goat serum (Zhongshan Jinqiao, China) was used for blocking, followed by incubation at room temperature for 60 min. After the serum was removed, an anti-MUC1 antibody (1:200, Boster, China) was added and incubated for 1 h at room temperature. Sections were washed with TBST three times for 5 min each. A five-color multifluorescent immunohistochemical staining kit (Absin, China) was used, with the secondary antibody from the kit (Absin, China) added according to the manufacturer's instructions, and incubated at room temperature for 10 min, followed by three washes with TBST for 5 min each. Fluorescent dye 620 from the kit (1:100, Absin, China) was added, incubated at room temperature for 10 min, and washed with TBST for 5 min each. The slices were again subjected to microwave heating, protected from light, and washed with phosphate buffer three times for 5 min each after natural cooling to room temperature. Goat serum was reapplied, and the mixture was incubated at room temperature for 60 min. The serum was removed, and anti-SIGLEC9 (1:200, Proteintech, China) was added, with incubation at 4 °C overnight. The following day, the sections were brought to room temperature for 1 h and washed three times with TBST for 5 min each. The secondary antibody from the kit (Absin, China) was then applied, incubated for 10 min at room temperature in the dark, and washed three times with TBST for 5 min each. Finally, 520 fluorescent dyes (1:100, Absin, China) were added, incubated at room temperature for 10 min in the dark, and washed with TBST for 5 min each. DAPI from the kit (1:100, Absin, China) was then added and incubated at room temperature for 10 min in the dark, followed by washing with TBST for 2 min. This was repeated three times. Finally, images were captured using a Nikon confocal AX nsparc (Nikon, Japan) after the antiquenching agent was added to the tablet. The mean gray values (mean) of MUC1 (red) and SIGLEC9 (green) were analyzed using a color image analysis system (FIJI 2.9.0) (Mean = Integrated density (IntDen) / area). The relative fluorescence intensity of the two groups was calculated using Version 9.3.1 software (GraphPad Software) (relative fluorescence intensity of cervical cancer group = mean of cervical cancer group/average mean of control group; relative fluorescence intensity of normal control group = mean of normal control group/average mean of control group).

### Cell immunofluorescence

For immunofluorescence staining, the cells were first fixed with 4 % paraformaldehyde, followed by incubation with drops of hydrogen peroxide to inhibit endogenous peroxidase activity. The cells were incubated with goat serum (Zhongshan Jinqiao) for 60 min at room temperature. Cells were labeled overnight with MUC1(Boster, 1:200 dilution) at 4 °C overnight followed by coralite488-conjugated goat anti-rabbit IgG (*H* + *L*) (Proteintech, 1:1000 dilution) for 1 h at 37 °C. Finally, the cells were counterstained with DAPI and observed under a confocal microscope. The mean gray values (mean) of MUC1 (red) were analyzed using a color image analysis system (FIJI 2.9.0) (Mean = Integrated density (IntDen) / area). The relative fluorescence intensity of the four groups was calculated using Version 9.3.1 software (GraphPad Software).

### Four-color multiplex immunohistochemistry

FFPE slides were baked for 4 h, deparaffinized with dimethylbenzene, and dehydrated using an ethanol gradient. The sections were then placed in Tris-EDTA antigen repair solution (pH 9.0, Solarbio, China) and subjected to microwave heating at 95 °C for 15 min to facilitate antigen retrieval. Following natural cooling to room temperature, the sections were washed three times with phosphate buffer for 5 min each. Goat serum (Zhongshan Jinqiao, China) was used for blocking, followed by incubation at room temperature for 60 min. After the serum was removed, the anti-CD4 (1:200, Abcam, USA) was added and incubated for 1 h at room temperature. Sections were washed with TBST three times for 5 min each. A five-color multifluorescent immunohistochemical staining kit (Absin, China) was used, with the secondary antibody from the kit (Absin, China) added according to the manufacturer's instructions, and incubated at room temperature for 10 min, followed by three washes with TBST for 5 min each. Fluorescent dye 700 (1:100, Absin, China) was added, incubated at room temperature for 10 min, and washed with TBST for 5 min each. The slices were then subjected to microwave heating, protected from light, and washed three times with phosphate buffer for 5 min each after natural cooling to room temperature. Goat serum was reapplied, and the mixture was incubated at room temperature for 60 min. After removing the serum, anti-SIGLEC9 (1:100, Proteintech, China) was added and incubated at 4 °C overnight. The following day, the sections were allowed to reach room temperature for 1 h and then washed three times with TBST for 5 min each. The secondary antibody from the kit (Absin, China) was then applied, incubated for 10 min at room temperature in the dark, and washed three times with TBST for 5 min each. Finally, 620 fluorescent dyes (1:100, Absin, China) were added, incubated at room temperature for 10 min in the dark, and washed with TBST for 5 min each. The slices were subjected to microwave heating once more, shielded from light, and washed with phosphate buffer three times for 5 min each after natural cooling to room temperature. Goat serum was added and incubated at room temperature for 60 min. After the serum was removed, the anti-MUC1 (1:200, Boster, China) was added and incubated for 1 h at room temperature. Sections were washed with TBST three times for 5 min each. The secondary antibody from the kit (Absin, China) was added and incubated at room temperature for 10 min in the dark, after which the membrane was washed three times with TBST for 5 min. Subsequently, 520 fluorescent dyes (1:100, Absin, China) were added and incubated at room temperature for 10 min in the dark, followed by washing with TBST for 5 min. This was repeated five times. DAPI from the kit (1:100, Absin, China) was then added and incubated at room temperature for 10 min in the dark, followed by washing with TBST for 2 min. This was repeated three times. Finally, images were captured using a Nikon confocal AX nsparc microscope (Nikon, Japan) after the antiquenching agent was added to the tablet.

### Flow cytometry (FCM)

SIGLEC9^+^ T-cells in single cell suspension were analyzed with PCP5.5-CD3 (Biolegend, USA, 300,328), APC—Cy™7-CD4 (Biolegend, USA, 317,450), FITC—CD8 (BD, USA, 551,347), PE-SIGLEC9 (Biolegend, USA, 351,504); SIGLEC9^+^ M1/M2 in single-cell suspension were analyzed with PE-CD14 (Biolegend, USA, 325,634), PE/Cyanine7-CD68 (Biolegend, USA, 333,816), AntibodyAlexa Fluor-CD86 (Biolegend, USA, 305,414), PE/Cyanine5 -CD206 (Biolegend, USA, 321,108). Single-cell suspensions were prepared by gently pressing the tissues through a sterile 300-mesh nylon filter.

### Statistical analysis

GraphPad version 9.3.1 software was used to analyze the data. The data are expressed as the mean ± SD. Comparisons between groups were performed using unpaired Student’s *t*-tests or analysis of variance. Statistical significance was defined as *p* < 0.05. Significance levels are indicated as follows: * *p* < 0.05, ** *p* < 0.01, *** *p* < 0.001, and **** *p* < 0.0001.

## Results

### Expression of the SIGLEC9 in patients with cervical cancer

**Fig. 1:**
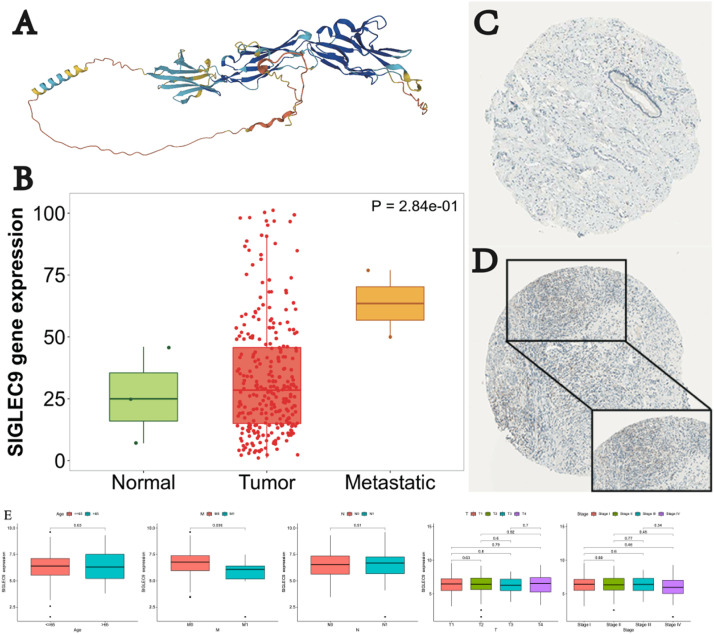
SIGLEC9 was up regulated in cervical cancer. (A) The Protein Structures of SIGLEC9 from the Alphafold pretein structure database. (B) The expression of SIGLEC9 gene in cervical cancer from TNMplot database. (C‒D) The protein expression of SIGLEC9 was obtained from the Human Protein Atlas. (E) Association of SIGLEC9 mRNA expression and different Age, TNM and pathological stages in patients with different cancers from TCGA.

To investigate the potential role of SIGLEC9 in cervical cancer, the authors first analyzed its protein structure using the Alphafold Protein Structure Database (Fig. 1A). Subsequently, the authors compared the expression levels of SIGLEC9 in normal and cancer tissues through the TNMPlot database. The findings revealed a trend of increased expression of SIGLEC9 in cancer tissues, although this increase was not statistically significant (Fig. 1B). To further investigate whether this change in expression is specific to epithelial cells, the authors examined the IHC data from the Human Protein Atlas database. The representative IHC data illustrated a notable downregulation of SIGLEC9 in normal tissues, contrasting with an increase in cancer tissues (Fig. 1C‒D). Consequently, these findings collectively suggest that SIGLEC9 is overexpressed in cervical cancer tissues.

TCGA-CESC was selected as the study cohort to investigate the correlation between SIGLEC9 expression and clinicopathologic staging. Patients with CESC were categorized into SIGLEC9-low expression and SIGLEC9-high expression groups using the median expression value of SIGLEC9. Subsequently, the authors analyzed the relationship between the SIGLEC9-high and -low expression groups and the clinical indicators of CESC in the TCGA cohort. The results illustrated a notable disparity between the SIGLEC9 high and low expression groups in relation to tumor M stage (Fig. 1E).

### Functional analysis of the SIGLEC9 in patients with cervical cancer

**Fig. 2:**
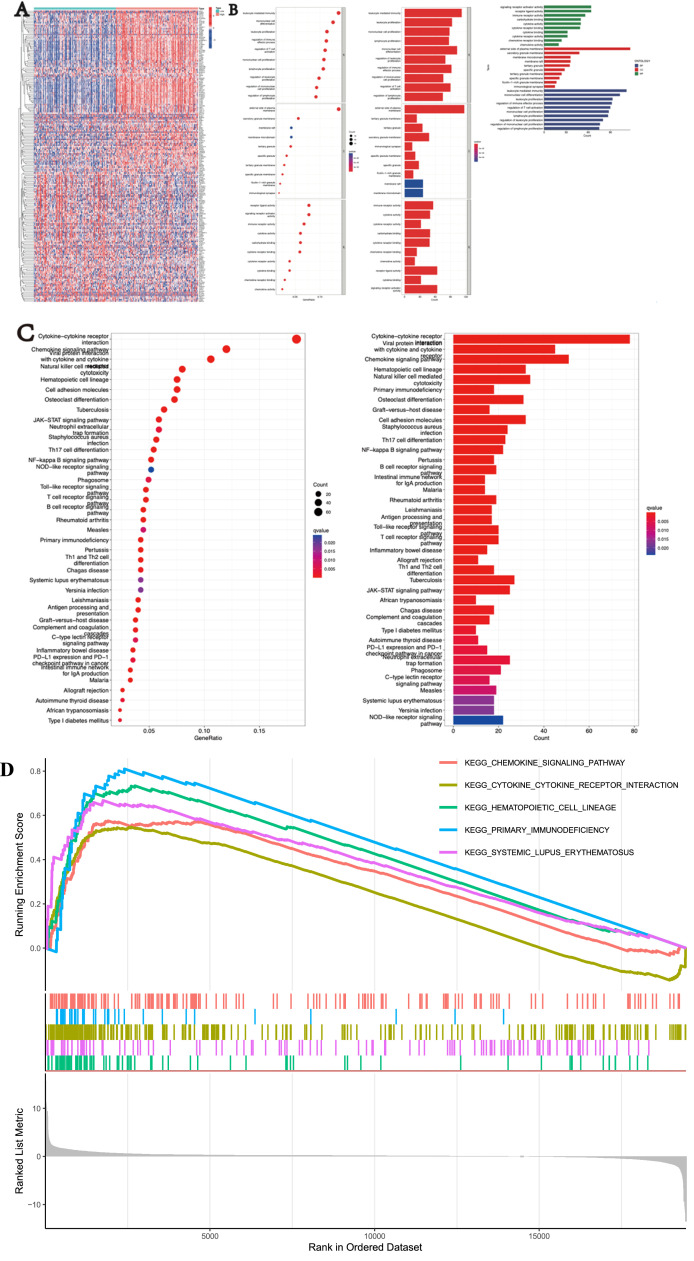
Functional enrichment analysis with The Cancer Genome Atlas (TCGA). (A) DEGs between the two groups. (B) GO analysis. (C) KEGG analysis. (D) GSEA analysis results.

The authors further investigated the potential biological functions and pathways of SIGLEC9 in different risk groups. GO enrichment and KEGG pathway analyses were performed on Differentially Expressed Genes (DEGs) between high-risk and low-risk groups (Fig. 2A). The GO analysis revealed that biological processes were associated with signaling receptor activator activity, cellular components were enriched in the external side of the plasma membrane, and molecular functions were related to leukocyte-mediated immunity (Fig. 2B). KEGG analysis highlighted pathways such as cytokine-cytokine receptor interaction, chemokine signaling, and viral protein interactions with cytokines and cytokine receptors (Fig. 2C). Additionally, Gene Set Enrichment Analysis (GSEA) was utilized to explore the biological functions of SIGLEC9 in cervical cancer (Fig. 2D). These findings suggest that SIGLEC9 may play a crucial role in cellular interactions.

### Analysis of SIGLEC9-related tumor microenvironment in patients with cervical cancer

**Fig. 3:**
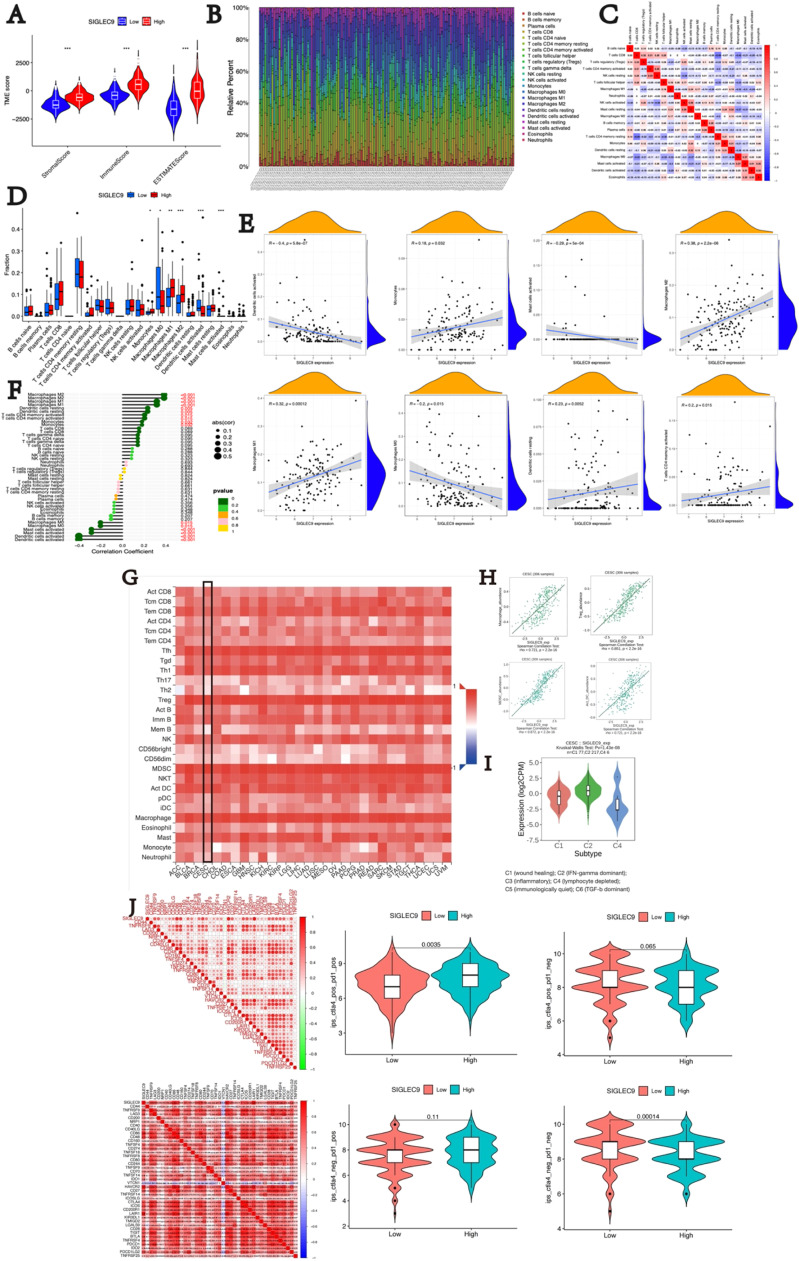
Correlation analysis between SIGLEC9 and immune microenvironment in TCGA and TISIDB. (A) Violin plots comparing StromalScore, ImmuneScore, ESTIMATEScore between high and low expression of SIGLEC9, respectively. (B) The proportion of immune cell types in all patients between high- and low-SIGLEC9 groups. (C) Correlation matrix of immune cell proportions. (D) Differences in immune cell infiltration between the high and the low expression of SIGLEC9, respectively. (E) Scatter plot of the relationship between high and low SIGLEC9 expression and the level of immune cell infiltration. (F) Lollipop plot of SIGLEC9 expression in relation to immune cells. (G) Spearman correlations between expression of SIGLEC9 and TILs across CESC. (H) The TISIDB database to analyzed SIGLEC9 expression in macrophages, MDSC, Act DC and Treg cells. (I) SIGLEC9 mRNA expression in different immune subtypes in CESC via TISIDB. (J) Relationship between SIGLEC9 expression and immune checkpoints. * *p* < 0.05, ** *p* < 0.01, *** *p* < 0.001, and **** *p* < 0.0001.

The tumor microenvironment is composed of various cell types, including tumor cells, stromal cells, fibroblasts, and immune cells. This study explored the relationship between the immune microenvironment and SIGLEC9 expression levels in cervical cancer. The ESTIMATE algorithm was used to compare microenvironment scores between high and low SIGLEC9 expression groups. Results in Fig. 3A showed that the group with high SIGLEC9 expression had significantly higher stromal cell and immune scores compared to the low expression group (*p* < 0.001). To further investigate the association between SIGLEC9 and tumor-infiltrating immune cells, various immune invasion algorithms were employed to assess immune cell infiltration levels. Initially, RNA-seq datasets from cervical cancer patients were analyzed using the CIBERSORT algorithm to examine the landscape of immune cell infiltration (Fig. 3B‒C). The boxplot analysis revealed that the SIGLEC9-high group had significantly lower proportions of M0 macrophages and activated dendritic cells. In contrast, this group showed significantly higher levels of monocytes, M1 macrophages, and M2 macrophages (Fig. 3D). Scatter plots indicated a positive correlation between SIGLEC9 expression and the numbers of M1 macrophages, M2 macrophages, monocytes, and resting dendritic cells, while demonstrating a negative correlation with M0 macrophages, activated mast cells, and activated dendritic cells (Fig. 3E). Subsequently, a lollipop plot was created based on the aforementioned data (Fig. 3F). These results suggest that SIGLEC9 expression is most strongly associated with macrophage M2 populations in the tumor microenvironment.

Furthermore, the authors use the TISIDB database to analyze the expression of SIGLEC9 and TILs across CESC. The findings revealed a strong correlation between SIGLEC9 expression and macrophages, MDSC, Act DC, and Treg cells (Fig. 3G). Scatter diagrams further illustrated the positive correlation between SIGLEC9 and the aforementioned immune cell types, consistent with the TCGA database (Fig. 3H). By employing molecular typing of immune subtypes, the authors explored SIGLEC9 mRNA expression across different immune subtypes. Notably, a distinct pattern of SIGLEC9 expression was observed in C1 (wound healing), C2 (IFN-γ dominant), C3 (inflammatory), C4 (lymphocyte deplete), C5 (immunologically quiet), and C6 (TGF-β dominant) subtypes in CESC, with the highest expression in subtype C2(Fig. 3I). This differential expression of SIGLEC9 in immune subtypes may help elucidate its varied roles in cancer prognosis. Overall, these results confirm the association of the SIGLEC9 gene with immune cells in the immune microenvironment, particularly macrophages and CD4+ T-cells.

To further investigate the role of SIGLEC9 in cervical cancer-induced immune responses, the authors conducted a Pearson correlation analysis using the TCGA dataset to assess the relationship between SIGLEC9 and checkpoint members in tumor-induced immune responses. The present results, depicted in Fig. 3J and Supplementary Table S1, demonstrated a strong correlation between SIGLEC9 and LAIR1 (Cor = 0.93, *p* < 0.001), HAVCR2 (Cor = 0.896, *p* < 0.001), and CD86 (Cor = 0.861, *p* < 0.001). Interestingly, the authors also observed significant correlations between SIGLEC9 and other checkpoint members such as CD274 (PD-L1), CTLA4, LAG3, ICOS, CD27, CD86, and CD48 (Fig. 3J). These findings suggest that SIGLEC9 may influence anti-tumor immune responses by co-regulating with various immune checkpoint molecules, supporting the potential use of combination cancer immunotherapy targeting these molecules in future studies. The present results imply that combination treatment may be particularly effective in patients with high levels of SIGLEC9.

### Single-cell analysis of tumor tissue and normal tissue in patients with cervical cancer

**Fig. 4:**
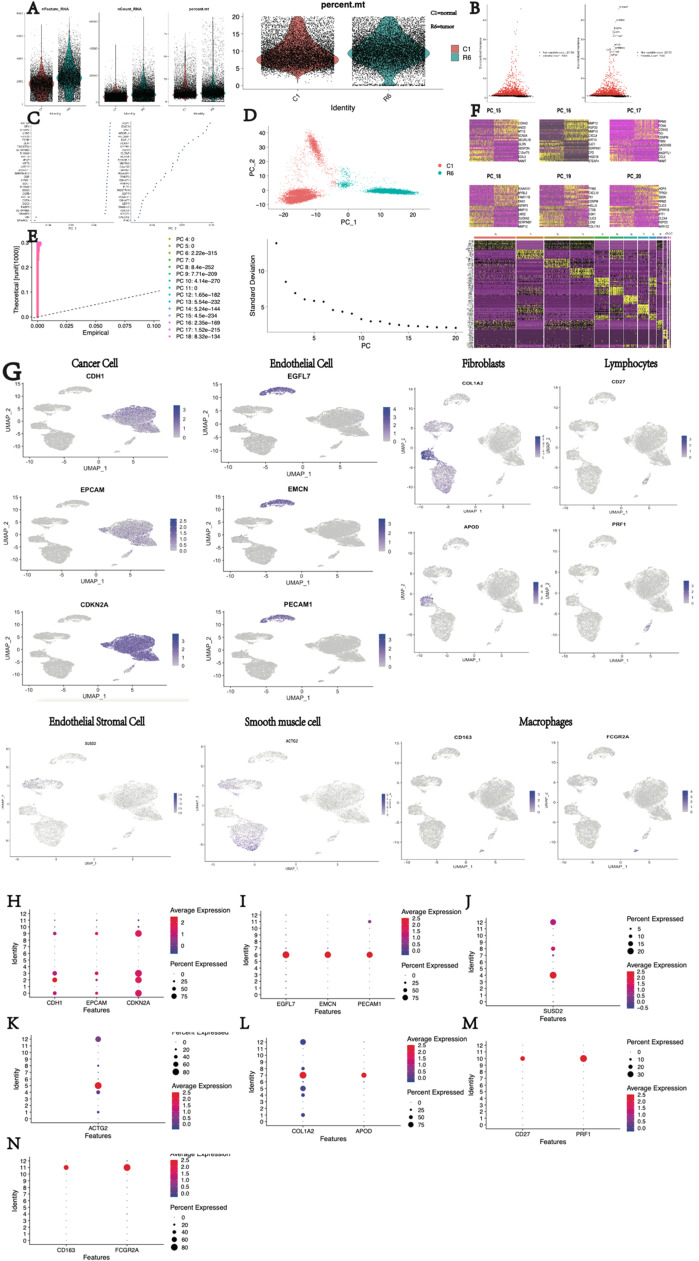

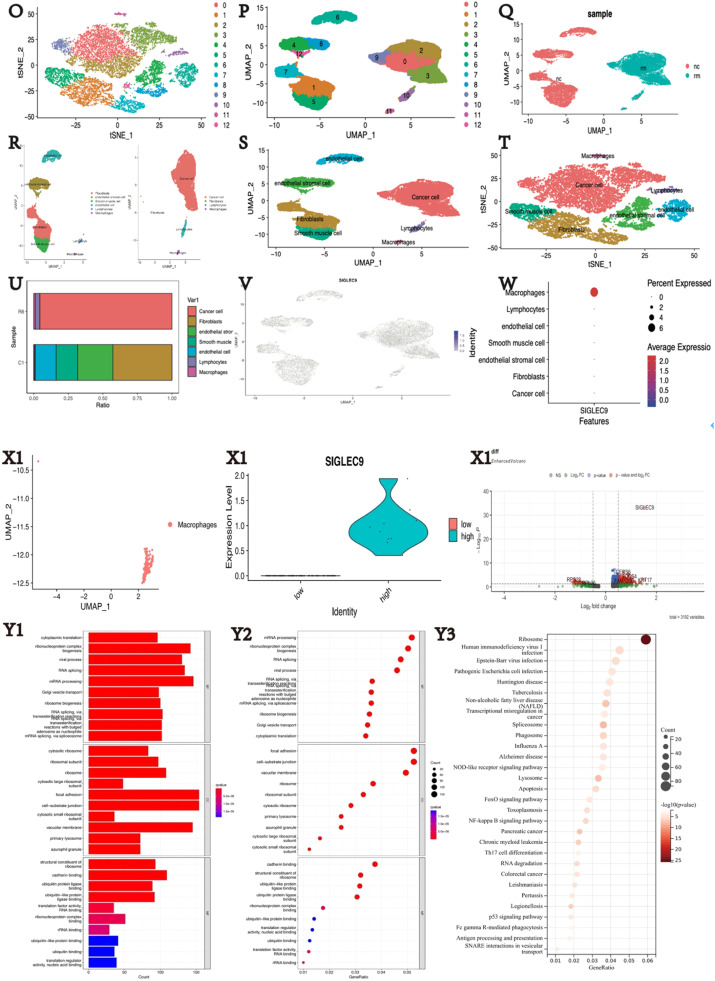
Differential expression analysis and cell subset identification in cervical cancer using single-cell analysis. (A‒B) Stringent cell filtration criteria were applied, including the selection of cells with a gene count between 200 and 5000 (nFeature > 200 and ≤ 5000) and a limited proportion of mitochondrial genes (percent.mt < 10). (C‒F) Principal Component Analysis was performed. (G) Expression profiles of well-known markers were assessed across different cell types in the central nervous system (CC). (H‒N) Bubble plot of marker gene expression in the identification of different cell types. (O‒P) The uniform manifold approximation and projection and the t-distributed Stochastic Neighbor Embedding (t-SNE) plot demonstrating main cell types in CC. (Q‒R) Exhibition of group (nc = normal, rm = tumor). (S‒T) Exhibition of cell subsets. (U) Distribution of cells in each sample. (V) Comparison of SIGLEC9 expression between tumor tissue and adjacent normal tissue. (W) Differential expression of SIGLEC9 between tumor tissue and normal tissue. (X1) The UMAP of macrophages. (X2) Macrophages into two groups with high and low SIGLEC9 expression. (X3) DEGs between high- and low-SIGLEC9 groups in macrophages. (Y1‒2) Gene Ontology analysis between high- and low-SIGLEC9 groups in macrophages. (Y3) Kyoto Encyclopedia of Genes and Genomes analysis between high- and low-SIGLEC9 groups in macrophages.

Based on the results above, the authors found that SIGLEC9 exhibits the strongest association with macrophages. To further explore this correlation, the authors performed single-cell analysis. Single-cell data were normalized and pooled from all samples, with low-quality cells filtered out (Fig. 4A‒B). Following gene expression normalization, Principal Component Analysis (PCA) was used for dimensionality reduction (Fig. 4C‒F). The authors merged the tumor and normal samples to perform unsupervised clustering to identify distinguished cell populations. Seurat v3.0 with default parameters was utilized in this study to classify different cell subsets based on typical marker genes (Fig. 4G). Seven types of cells were mainly identified, including cancer cells (CDH1, EPCAM, CDKN2A), endothelial cells (EGGL7, EMCN, PECAM1), endometrial stromal cells (SUSD2), fibroblasts (COL1A2, APOD), lymphocytes (CD28A, CD27, PRF1), macrophages (CD163, FCG2A), and smooth muscle cells (ACTG2) (Fig. 4H‒N). Cell lineages were further categorized based on canonical marker gene expression (Fig. 4S‒T). The proportion of cell types in each sample was calculated, revealing significantly higher lymphocyte proportions in tumor samples compared to normal samples, and higher macrophage proportions in tumor samples (FigureU). Subsequent analysis focused on macrophages in tumors, with the expression of SIGLEC9 in various cell subsets between tumor and normal tissues examined (Fig. 4V). SIGLEC9 was identified as a potentially important regulator of macrophages (Fig. 4W).

Macrophages were isolated for analysis, as shown in Fig. 4X1. Based on SIGLEC9 expression levels, the macrophages were divided into two groups: high and low SIGLEC9 expression, as illustrated in Fig. 4 × 2. Subsequently, differential genes between these two groups were identified (Fig. 4× 3). GO enrichment analysis revealed that these differential genes were significantly enriched in pathways such as mRNA processing (Fig. 4Y1‒2). Furthermore, KEGG enrichment analysis showed that the differentially expressed genes were enriched in pathways related to Fc gamma R-mediated phagocytosis and antigen processing and presentation (Fig. 4Y3). This suggests that the differential genes identified in the two groups are involved in antigen presentation pathways, indicating a relationship between SIGLEC9 gene expression and the antigen processing ability of macrophages.

### SIGLEC9 interaction network

**Fig. 5:**
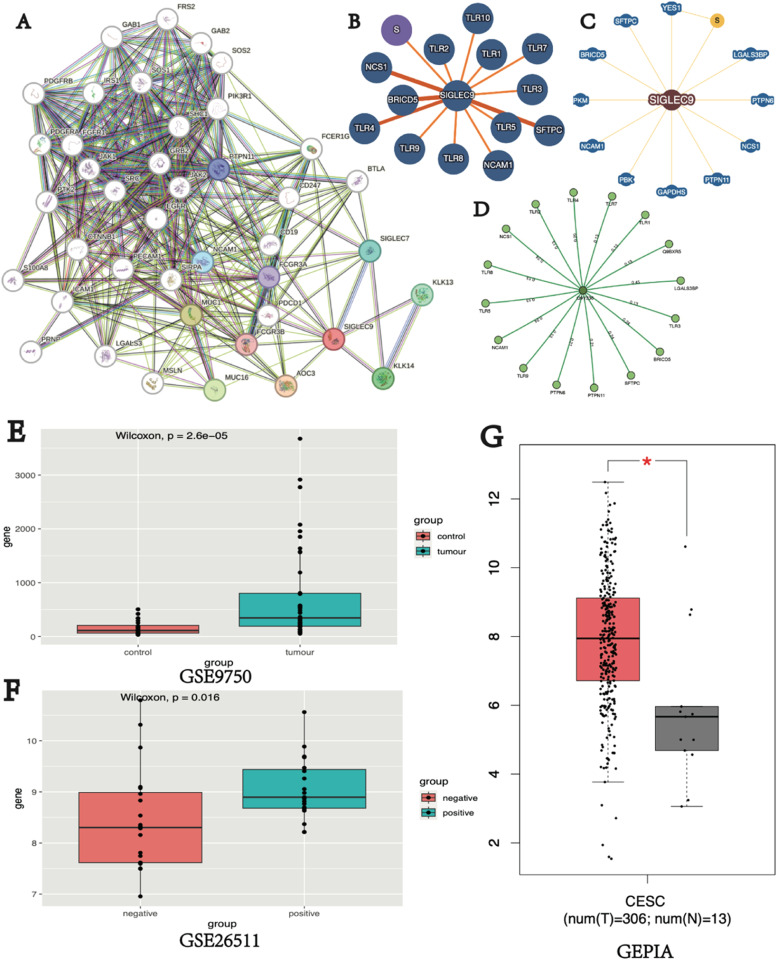
PPI map of proteins interacting with SIGLEC9. (A‒D) The PPI network of SIGLEC9 from STRING (A), IntAct (B), BioGIRD (C), Mentha (D). (E) The expression of MUC1 gene in cervical cancer and normal cervical tissue from GEO database. (F) Expression of MUC1 gene in cervical cancer with or without lymph node metastasis from GEO database. (G) The expression of MUC1 gene in cervical cancer and normal cervical tissue from GEPIA database (* *p* < 0.05).

Proteins interact in various ways to create a complex Protein-Protein Interaction (PPI) network that regulates cell functions. Therefore, it is essential to analyze the structure and role of protein complexes by establishing PPI patterns. To study the proteins interacting with SIGLEC9, the authors utilized STRING (Fig. 5A), IntAct (Fig. 5B), BioGIRD (Fig. 5C), and Mentha (Fig. 5D) databases. These findings indicate a protein association between SIGLEC9 and MUC1. Consequently, the authors examined the mRNA expression levels of MUC1 in cervical cancer and normal tissues using the GEO (Fig. 5E‒F) and the GEPIA (Fig. 5G) databases. These results demonstrated significantly higher expression levels of MUC1 in cervical cancer tissues.

### Validation of the expression level and clinical correlation of SIGLEC9 in cervical cancer

**Fig. 6:**
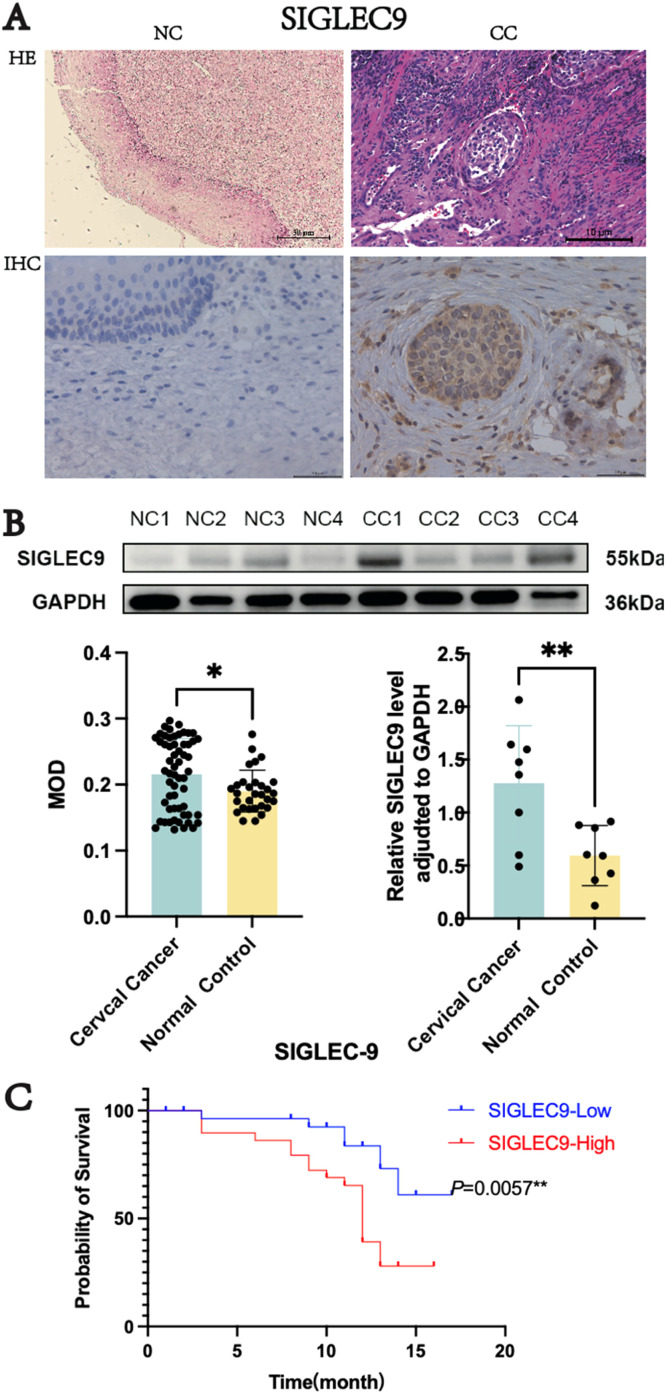
Expression level and clinical correlation in cervical cancer. (A) The expression of SIGLEC9 in cervical cancer patients (*n* = 58) and normal control (*n* = 30) with immunohistochemical staining. (B) The expression of SIGLEC9 in cervical cancer patients with western blotting (*n* = 8). (C) The cervical cancer patients with a high SIGLEC9 expression (*n* = 29) had a shorter survival probability than those patients with a low SIGLEC9 expression (*n* = 29) (*p* = 0.0057). * *p* < 0.05, ** *p* < 0.01.

In order to further verify the expression level of SIGLEC9 in cervical cancer, IHC and Western blotting were conducted to compare SIGLEC9 expression differences between cancer tissues and normal tissues. These results showed that SIGLEC9 was significantly upregulated in cervical cancer tissues (Fig. 6A‒B), which was consistent with the result of the previous bioinformatics analysis based on TCGA, GEPIA and GEO databases.

To further investigate the clinical relevance of SIGLEC9 in cervical cancer, patients were categorized into high-SIGLEC9-expression and low-SIGLEC9-expression groups based on median MOD values of SIGLEC9 IHC (Fig. 6A). Subsequent analysis revealed that high-SIGLEC9-expression patients had a higher probability of advanced stage compared to low-SIGLEC9-expression patients (*p* = 0.0011) (Table 1), suggesting a potential role of SIGLEC9 in clinical staging. Furthermore, using the K-M plotter, it was observed that patients with high SIGLEC9 expression had a significantly shorter survival probability than those with low SIGLEC9 expression (*p* = 0.0057) (Fig. 6C), indicating a correlation between SIGLEC9 expression levels and patient prognosis. These findings collectively suggest that SIGLEC9 may be involved in the pathogenesis, progression, prognosis, and immune response in cervical cancer.

**Table 1:** Clinical characteristics of SIGLEC9 expression in cervical cancer patients.

Index		SIGLEC9-Low	SIGLEC9-High	p-value
Age		29	29	0.3578
	< 60	20	24	
	≥ 60	9	5	
FIGO classification^a^				0.0011^b^
	I	18	5	
	II+III+IV	11	24	
Lymph node metastasis				0.3313
	No	25	21	
	Yes	4	8	
Tumor diameter				0.4309
	< 4 cm	17	13	
	≥ 4 cm	12	16	
Vascular invasion				0.5301
	No	21	24	
	Yes	8	5	

a *p* ≤ 0.05,.

b *p* ≤ 0.01, *** *p* ≤ 0.001, **** *p* ≤ 0.0001.

### Validation interaction of SIGLEC9 with T, macrophages cell and MUC1

**Fig. 7:**
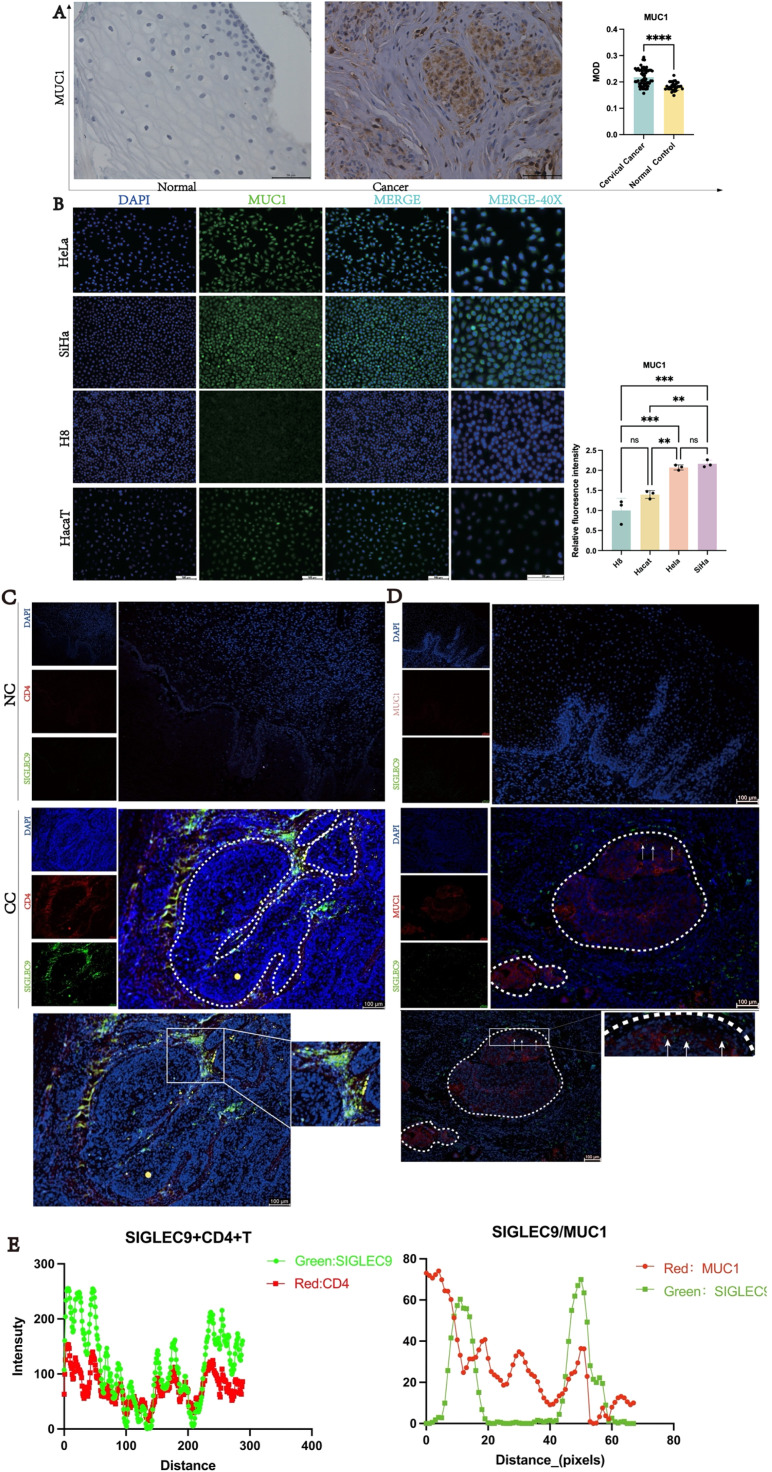

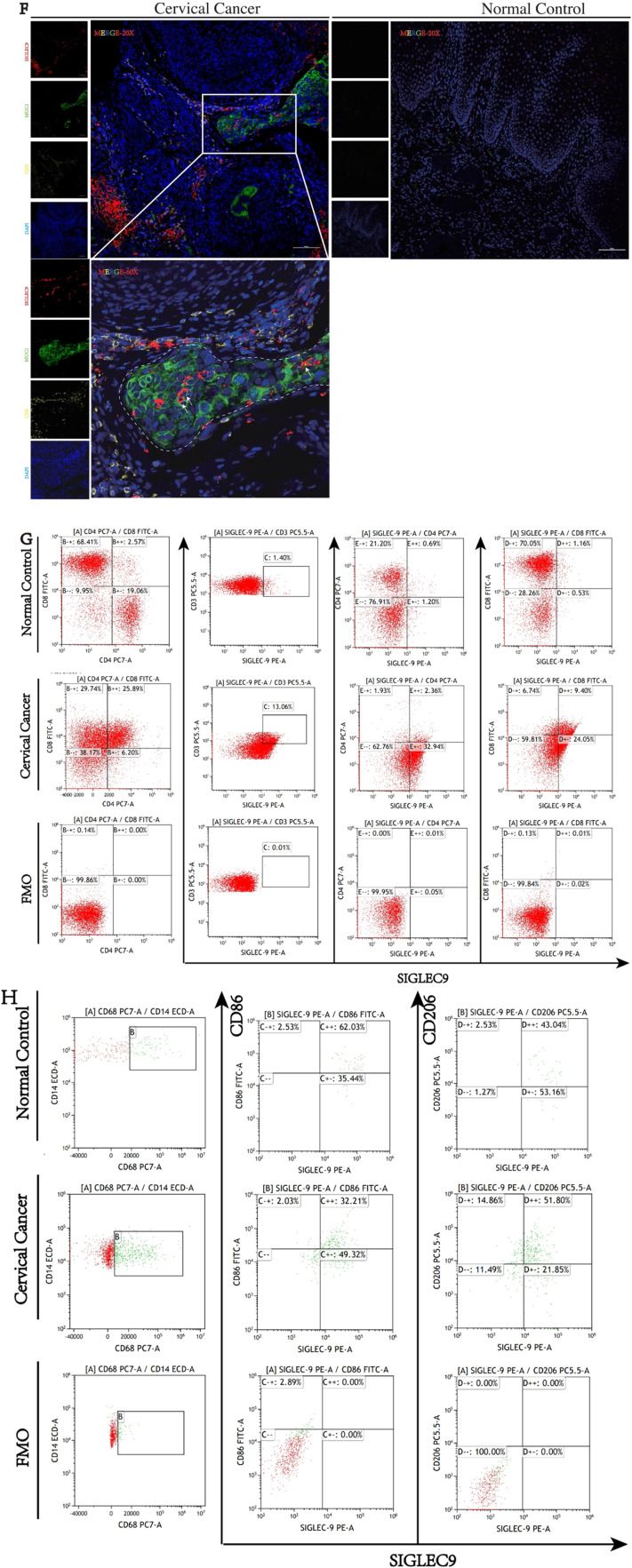

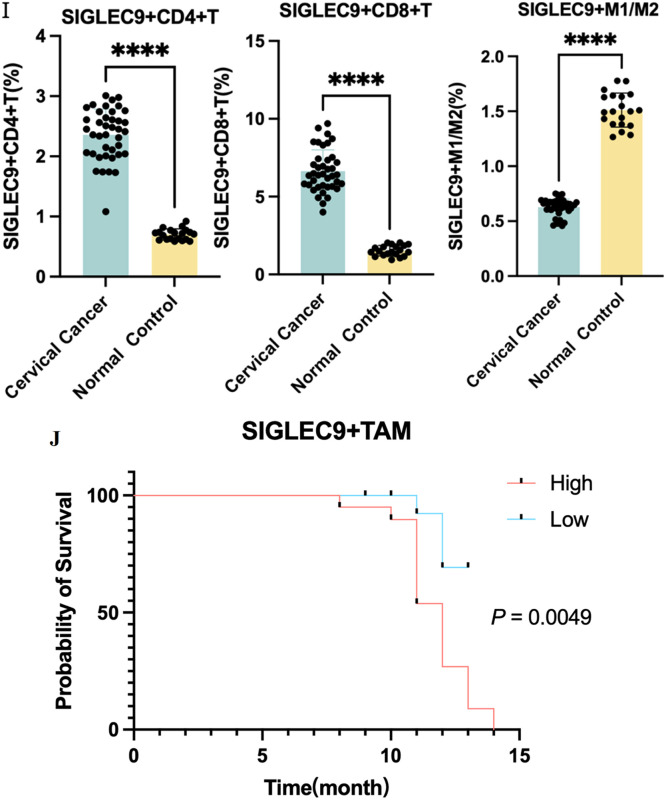
Interaction of SIGLEC9 with CD4+ T-cell and MUC1. (A) Immunohistochemical expression of MUC1 in cervical cancer and normal cervical tissues (*n* = 40). (B) MUC1 protein expression was analyzed by cell immunofluorescence (*n* = 3, three independent experiments). (C) The expression of SIGLEC9 (Green) and CD4 (Red), DAPI (Blue) in normal cervical tissue and in cancer tissues with double immunofluorescence. (D) Correlation between MUC1 (red) and SIGLEC9 (green), DAPI (Blue) in normal tissues (*n* = 6) and cancer tissues (*n* = 6). (E‒F) Multiplexed immunofluorescence for SIGLEC9 (Red), CD4 (Yellow), and MUC1 (Green), and DAPI (Blue) in cancer tissues and normal tissues. (G‒I) The flow cytometry expression of SIGLEC9+ CD4+ T/CD8+ T-cells and SIGLEC9+M1/M2 in cervical cancer patients (*n* = 40) and normal control (*n* = 20). ** *p* < 0.01, *** *p* < 0.001, and **** *p* < 0.0001. (J) The cervical cancer patients with a high SIGLEC9+ TAM cell infiltration (*n* = 20) had a shorter survival probability than those patients with a low SIGLEC9+ TAM cell infiltration (*n* = 20; *p* = 0.0049).

To elucidate the interaction of SIGLEC9 with T-cell and MUC1, firstly, the authors used IHC and IF to verify MUC1 in patients with cervical cancer, and cells were upregulated (Fig. 7A‒B). Then, the authors used dual immunofluorescence to verify the proliferation of SIGLEC9^+^CD4^+^T-cells (Fig. 7D) and SIGLEC9/MUC1 in cervical cancer tissue (Fig. 7C‒E). As shown in Fig. 7F, SIGLEC9^+^CD4^+^T-cells infiltrated around the tumor nests, and MUC1 was expressed in the tumor nests. To further verify whether SIGLEC9^+^CD4^+^T-cells interact with MUC1, the authors used a multiplex immunofluorescence technique to detect the infiltration of SIGLEC9^+^CD4 ^+^T-cells around the cancer nests (Fig. 7F). Taken together, these data confirm that MUC1 secreted by tumor cells interacts with SIGLEC9 on CD4^+^ T-cells in the tumor microenvironment of cervical cancer.

In addition, the authors collected single-cell suspension from cervical cancer tissues and normal controls, and detected the proportion of SIGLEC9+ CD4^+^T-cells, SIGLEC9+ CD8^+^T-cells and SIGLEC9+ M1 macrophages and SIGLEC9+ TAMs by flow cytometry. The authors confirmed that SIGLEC9+ CD4^+^T, SIGLEC9^+^CD8^+^T-cells and SIGLEC9+ M2 macrophages in cervical cancer tissues were higher than those in normal controls (Fig. 7G‒I).

In the previous studies, SIGLEC9 was found to be predominantly expressed on macrophages. Therefore, the authors conducted further research to investigate the impact of SIGLEC9+ TAMs on the prognosis of cervical cancer patients. The present analysis revealed that a high level of infiltration of SIGLEC9+ TAMs was significantly associated with decreased overall survival rates (*p* = 0.0049, Fig. 7J, Table 2). Additionally, SIGLEC9+ TAMs high infiltration served as an independent prognostic marker for overall survival, even after adjusting for age, tumor size, FIGO staging, histological type, and lymph node invasion (Table 3). To summarize, a high presence of SIGLEC9+ TAMs was correlated with poorer survival outcomes in cervical cancer.

**Table 2:** Patients clinical characteristics and relationship with SIGLEC9+ TAM infiltration.

Characteristics	Cases	SIGLEC9+ TAM cell infiltration		p-value
		Higher expression (> median)	Lower expression (≤ median)	
Age (years)	40	20	20	0.527
≤ 44	20	9	11	
> 45	20	11	9	
Tumor Size				0.001^c^
≤ 4 cm	22	6	16	
> 4 cm	18	14	4	
FIGO Stage				<0.0001^d^
Ⅰ	16	2	14	
Ⅱ/Ⅲ/IV	24	18	6	
Histological type				0.705
SCC	31	15	16	
ASCC	9	5	4	
LN metastasis				0.008^b^
Absent (-)	31	12	19	
Present (+)	9	8	1	

* *p* < 0.05,.

b *p* < 0.01,.

c *p* < 0.001,.

d *p* < 0.0001.

**Table 3:** Univariate and multivariate analysis for living state.

Variables	Living State		Univariate	Multivariate			
			p-value	p-value	HR	95 % CI	
	Live	Death				Low	Up
Age (years)			0.744	0.258	0.348	0.056	2.167
≤ 44	12	8					
> 45	13	7					
Tumor Size			0.14	0.411	0.399	0.045	3.564
≤ 4 cm	16	6					
> 4 cm	9	9					
FIGO Stage			0.008^b^	0.118	8.171	0.585	114.148
Ⅰ	14	2					
Ⅱ/Ⅲ/IV	11	13					
Histological type		0.625	0.925	0.908	0.123	6.687	
SCC	20	11					
ASCC	5	4					
LN metastasis			0.006^b^	0.343	2.728	0.343	21.696
Absent (-)	21	10					
Present (+)	4	5					
SIGLEC9+TAM			0.003^b^	0.036^a^	7.452	1.144	48.551
High	8	12					
Low	17	3					

a *p* ≤ 0.05,.

b *p* ≤ 0.01, *** *p* ≤ 0.001, **** *p* ≤ 0.0001.

## Discussion

This study found that SIGLEC9 is highly expressed in cervical cancer and that its elevated expression is positively correlated with poor prognosis. Additionally, the study revealed that SIGLEC9 is predominantly expressed on macrophages and T-cells, with high levels of SIGLEC9+ TAMs and SIGLEC9+ T-cells observed in cervical cancer tissues. Furthermore, the research suggests that SIGLEC9+ TAMs may serve as a potential prognostic biomarker for cervical cancer. The study indicates that MUC1, secreted by cervical cancer cells, interacts with SIGLEC9 on macrophages and T-cells, promoting TAMs differentiation and inhibiting T-cell function, ultimately contributing to immune evasion in cervical cancer. These findings offer valuable insights into potential immunotherapeutic strategies for cancer, although further studies are required to explore this mechanism in greater depth.

Cervical cancer ranks as the fourth most common cancer among women. In 2020, there were an estimated 604,000 new cases of cervical cancer, leading to 342,000 deaths globally.^19^ The integration of Human Papillomavirus (HPV) in cervical cancer triggers complex interactions with immunosuppressive phenotypes and impaired immune surveillance.^20^ The research conducted by Douglas Hanahan and his group indicates that the new vaccine formulation, combined with local immune stimulation and standard chemotherapy, holds promise for further improving treatment outcomes for patients with HPV-related cancers.^21^^,^^22^ In this context, immunotherapy has emerged as a promising treatment option, as evidenced by the recent approval of programmed cell Death Protein 1 (PD-1) blocking antibodies for the treatment of recurrent or metastatic cervical cancer. In 2021, the US Food and Drug Administration (FDA) approved pembrolizumab for use in recurrent and advanced cervical cancer.^23^ Additionally, the European Medicines Agency (EMA) approved cemiplimab, a PD-1-specific antibody, in 2022, due to its significant improvement in overall survival in cervical cancer.^24^ However, despite the use of Immune Checkpoint Blockers (ICBs), the clinical Objective Response Rate (ORR) for ICBs in practice rarely exceeds 20 %.^25^ To effectively address this issue, the integration of treatment strategies has become crucial for improving patient outcomes. In this context, recognizing the critical role of SIGLEC9 is essential.

Sialic acid-binding Immunoglobulin-like Lectins (SIGLECS) are a new type of immune checkpoint that plays a role in tumor immunosuppression. They could potentially serve as new targets, biomarkers, or prognostic factors for immunotherapy.^26^^,^^27^ Siglecs are a family of single-pass cell surface receptors characterized by an N-terminal domain that binds sialylated glycans. Most SIGLECS have one or multiple Immunoreceptor Tyrosine-based Inhibitory Motifs (ITIM) on the C-terminus that trigger inhibitory signals through the recruitment of tyrosine and inositol phosphatases. SIGLECS are predominantly found on immune cells, with each cell expressing a unique combination of SIGLECS that allows them to respond to distinct sialylation patterns.^28^ Among the Siglecs, SIGLEC9 (CD329) is a promising immune checkpoint target that, when blocked, enhances the body's anti-cancer immune response.^6^^,^^29^ Previous studies have shown that siglec-9 is highly expressed in ovarian cancer,^7^ pancreatic cancer cells[12] and colon cancer.^11^ This study comprehensively analyzed the expression pattern of SIGLEC9 in TMN Plot databases. SIGLEC9 exhibited an increasing trend in the cancer and metastasis groups, although the statistical significance was not reached, possibly due to the small sample size of the normal and metastasis groups. Immunohistochemical images from the HPA database revealed that the staining intensity of SIGLEC9 was moderate in cervical cancer tissues and weak in normal cervical tissues. Furthermore, protein levels of SIGLEC9 were investigated in cervical cancer patients and normal tissues from myoma of uterus patients who screened negative for cervical cancer and underwent total hysterectomy at the First Affiliated Hospital of Xinjiang Medical University. Both IHC and Western blotting results indicated high expression of SIGLEC9 in cancer tissues. Additionally, SIGLEC9 expression was notably upregulated in tumor stage according to the TCGA database. The authors’ own database also demonstrated a correlation between high SIGLEC9 expression and advanced tumor stage in cervical cancer patients. Studies have suggested that high SIGLEC9 expression is associated with reduced survival in cancer patients,^30^ and the present findings supported this by showing that patients with high SIGLEC9 expression had a lower survival probability than those with low expression. These findings indicate that SIGLEC9 could serve as a diagnostic marker for poor prognosis in cervical cancer and may emerge as a promising target for immunotherapy in future treatment strategies for cervical cancer patients.

To further investigate the biological function of SIGLEC9, the authors performed Gene Ontology (GO), Kyoto Encyclopedia of Genes and Genomes (KEGG), and Gene Set Enrichment Analysis (GSEA), which collectively suggested that SIGLEC9 may play a critical role in cell-cell interactions. The study by Douglas Hanahan and colleagues found that squamous cell tumors of the cervix and skin, driven by Human Papillomavirus type 16 (HPV16), release four immunomodulatory factors ‒ IL-1α, IL-1β, IL-33, and IL-36β ‒ into the bloodstream. These factors skew the bone marrow toward granulocytic myeloid production, resulting in immunosuppressive neutrophils that accumulate in the spleen and tumors. These findings identify immunosuppressive myeloid cells in lymphoid organs as a mechanism by which HPV+ cancers circumvent tumor immunity, underscoring the need for targeted interventions to disrupt this process and enable the induction of effective antitumor immune responses.^31^^,^^32^ Subsequently, the authors utilized bioinformatics approaches to explore the potential mechanisms of SIGLEC9 within the Tumor Microenvironment (TME) of cervical cancer. Analysis using the ESTIMATE algorithm revealed that both stromal and immune scores were significantly higher in the high-SIGLEC9 group compared to the low-SIGLEC9 group. Given the complexity of tumor-infiltrating immune cells, which include various types such as Myeloid-Derived Suppressor Cells (MDSCs), neutrophils, macrophages, and regulatory T-cells (Tregs),^33^ the authors employed the CIBERSORT algorithm and the TISIDB database to assess the correlation between SIGLEC9 expression and immune cell composition in the TME. The results indicated that SIGLEC9 was most strongly correlated with macrophages. Furthermore, single-cell analysis validated that SIGLEC9 expression was notably higher in macrophages derived from tumor samples compared to normal tissues. The authors also observed a significant correlation between SIGLEC9 expression and macrophage antigen-processing capacity as well as their proliferative activity. Finally, flow cytometry confirmed that SIGLEC9+ TAMs were more abundant in cervical cancer tissue compared to normal cervical tissue, and high infiltration of SIGLEC9+ TAMs was associated with a poor prognosis in cervical cancer. Previous studies have demonstrated that the SIGLEC9 receptor influences macrophage differentiation, driving their transition toward an M2-like, immunosuppressive, and tumor-promoting phenotype.^7^^,^^12^ In the pancreatic cancer TME, sialic acid modulates monocytes to secrete IL-10 and IL-6, which subsequently activate the SIGLEC9 receptor, promoting the polarization and differentiation of monocytes into immunosuppressive TAMs.^12^ High infiltration of SIGLEC9+ TAMs has been associated with poor prognosis, and these cells may serve as biomarkers for prognosis assessment in colorectal cancer patients.^11^ The Omicron RBD enhances the binding of macrophages with SIGLEC-9, weakening the phagocytic and antigen-presenting functions of macrophages, thereby promoting immune evasion.^34^ Additionally, Wang et al.^7^ reported that elevated expression of SIGLEC9+ TAMs is linked to worse patient outcomes. Consistent with these findings, the analysis of cervical cancer data reveals that high expression of SIGLEC9^+^TAMs is an independent risk factor for poor prognosis. These results suggest that SIGLEC9+ TAMs could serve as a potential biomarker for prognostic evaluation in cervical cancer.

Douglas Hanahan and his group highlighted a connection between HPV-induced cancers, systemic amplification of myeloid cells, and the detrimental impact of myeloid cells on CD8^+^ T-cell activation and recruitment into the Tumor Microenvironment (TME).^31^^,^^32^ The authors used the CIBERSORT algorithm and TISIDB database found that SIGLEC9 was not only associated with macrophages, but also with T-cells. Hannah Egan et al.^35^ showed that the expression of sialyltransferase, α2, 3/6-chain sialic acid, and Siglec ligand in stromal cells increased under tumor conditions, which, combined with SIGLEC9+ T-cells to inhibit T-cell action to form an immunosuppressive tumor microenvironment. Therefore, the authors confirmed the high infiltration of SIGLEC9+ T-cells in cervical cancer tissues through dual IF and FCM. The results demonstrated significant infiltration of both SIGLEC9+ CD4^+^ T-cells and SIGLEC9+ CD8^+^ T-cells in the tumor microenvironment. In line with previous studies, these findings suggest that SIGLEC9+ T-cells could serve as a promising biomarker for the diagnosis of cervical cancer.

The present data suggest that high expression of SIGLEC9 is positively correlated with immune checkpoint genes such as LAG3, HAVCR2, CD86, and PD-1, implying that SIGLEC9 may contribute to the suppression of anti-tumor immune responses in cervical cancer by regulating these immune checkpoint molecules. Consistent with previous studies, which have demonstrated that combined treatment with anti-SIGLEC9 and anti-PD-1/PD-L1 enhances anti-tumor efficacy, these findings further support the potential role of SIGLEC9 in immune-based therapies for cervical cancer.^30^^,^^36^ Based on these results, the authors hypothesize that targeting both SIGLEC9 and PD-1 in PD-1-resistant cervical cancer patients could improve the immune response and potentially prolong survival. Future clinical trials are needed to validate the effectiveness of this combined therapeutic strategy and explore its potential as a new immune treatment option for cervical cancer patients.

To further investigate the function of SIGLEC9, the authors compiled genes that directly interact with SIGLEC9 from a protein interaction network database and constructed an interaction network module primarily consisting of SIGLEC9 genes. The network diagram revealed direct interactions between SIGLEC9 and MUC1, LGALS3BP, and CA125. Studies have shown that ligands binding to SIGLEC9 include Lectin Galactoside-Binding Soluble 3-Binding Protein (LGALS3BP).^37^ Studies by Richard Beatson et al.^38^ demonstrated that a sialylated tumor-associated glycoform of MUC1, known as MUC1-ST, can induce the differentiation of monocytes into Tumor-Associated Macrophages (TAMs) by engaging SIGLEC9. Additionally, research by Heinz Läubli et al.^37^ suggested that targeting SIGLEC9 interactions with tumor-associated ligands could be a promising therapeutic strategy to inhibit cancer progression. Furthermore, Sinyoung Jeong et al.^39^ proposed that MUC16 may bind to the SIGLEC9 receptor on Natural Killer (NK) cells, leading to the downregulation of NK cell cytotoxicity and enabling ovarian cancer cells to evade immune surveillance. The most SIGLEC9 ligand reported was MUC1.^38^^,^^40^ Therefore, the role of the MUC1/SIGLEC9 axis in cervical cancer deserves to be investigated. To validate these findings, the authors analyzed the expression pattern of MUC1 in cervical cancer using the GEO and GEPIA databases. Immunohistochemistry (IHC) and cell immunofluorescence confirmed increased MUC1 expression in cervical cancer tissues and cell lines. Lastly, multi-immunofluorescence staining revealed interactions between SIGLEC9+ T-cells and MUC1 in the tumor microenvironment.

However, it is important to recognize the limitations of this exploratory study. Given that the present research was based solely on bioinformatics analysis and partial clinical validation, without in vitro cell experiments or animal model testing, this study should be considered preliminary. Further experimental work and additional evidence are needed to fully elucidate the mechanisms by which SIGLEC9 regulates TAMs and T-cells.

## Conclusion

Overall, SIGLEC9 is highly expressed in cervical cancer and strongly correlates with FIGO staging and poor survival outcomes. Moreover, SIGLEC9 is expressed on TAMs and T-cells, where it interacts with MUC1 secreted by cervical cancer cells, facilitating immune evasion. These findings position SIGLEC9 as a promising target for cervical cancer immunotherapy, providing a potential new avenue for immune-based therapeutic strategies.

## Funding

This work was supported by National Natural Science Foundation of China (NSFC) under Grant / Award Number 82460502; National Natural Science Foundation of China (NSFC) under Grant / Award Number 82160545; Department of Gynecology, the First Affiliated Hospital of Xinjiang Medical University, State Key Laboratory of Pathogenesis, Prevention and Treatment of High Incidence Diseases in Central Asia under Grant /Award Number SKL-HIDCA-2024-GJ2.

## Authors’ contributions

Conception and design: Bihui Wang; Analysis and interpretation of the data: Yuejie Zhu, Zhenyu Ru; Drafting of the paper: Bihui Wang, Yulian Zhang, Pingfen Li, Liyuan Zhao, Manli Zhang, Mingkai Yu; Provided advice and technical assistance: Jianbing Ding; Revising it critically for intellectual content: Zhifang Chen. Final approval of the version to be published, and all authors agreed to be accountable for all aspects of the work.

## Data availability

The datasets generated and/or analyzed during the current study are available in the Cancer Genome Atlas (TCGA) repository, https://portal.gdc.cancer.gov/repository, and GEO repository, https://www.ncbi.nlm.nih.gov/geo/.

## Consent for publication

Not applicable.

## Ethics approval and consent to participate

Ethical approval (n° 20210226-18) was granted by the First Affiliated Hospital of Xinjiang Medical University (Xinjiang, China).

## Declaration of competing interest

The authors declare no conflicts of interest.

## Appendix Supplementary materials

Image, application 1

Image, application 2

Image, application 3

Image, application 4
