# Supplementary material for: The role of SIGLEC9 in immunosuppression and prognosis in cervical cancer
Source: Clinics (Sao Paulo). 2025 Dec 18;81:100849. doi: 10.1016/j.clinsp.2025.100849 (PMC12771336; doi:10.1016/j.clinsp.2025.100849)
Supplement: Supplementary file 1 [file mmc1.docx]

**CLINICS-D-24-00428_Supplementary Material**

**Supplementary Data Table S1** The expression correlation between SIGLEC9 and checkpoint members in tumor-induced immune response using Pearson correlation analysis with TCGA dataset.

**Supplementary Data Table S2** 20-known human STs (ST3GAL5 was not found in CESC database) in CESC tumors revealed differential expression of the ST enzymes.
