# Supplementary material for: The role of SIGLEC9 in immunosuppression and prognosis in cervical cancer
Source: Clinics (Sao Paulo). 2025 Dec 18;81:100849. doi: 10.1016/j.clinsp.2025.100849 (PMC12771336; doi:10.1016/j.clinsp.2025.100849)
Supplement: Supplementary file 2 [file mmc2.docx]

| Query | Gene | cor | pvalue |
| --- | --- | --- | --- |
| SIGLEC9 | CD44 | 0.22369887 | 8.35E-05 |
| SIGLEC9 | TNFRSF9 | 0.69337404 | 6.90E-45 |
| SIGLEC9 | LAG3 | 0.64533496 | 3.36E-37 |
| SIGLEC9 | CD200 | 0.32568853 | 6.08E-09 |
| SIGLEC9 | NRP1 | 0.37901584 | 8.03E-12 |
| SIGLEC9 | CD40 | 0.5162254 | 4.18E-22 |
| SIGLEC9 | CD40LG | 0.54087581 | 1.70E-24 |
| SIGLEC9 | CD86 | 0.86109486 | 1.03E-90 |
| SIGLEC9 | CD48 | 0.774639 | 4.70E-62 |
| SIGLEC9 | CD160 | 0.23130857 | 4.67E-05 |
| SIGLEC9 | TNFSF4 | 0.60493059 | 9.98E-32 |
| SIGLEC9 | CD274 | 0.55057258 | 1.71E-25 |
| SIGLEC9 | TNFSF18 | 0.26571007 | 2.62E-06 |
| SIGLEC9 | TNFRSF8 | 0.62234867 | 5.47E-34 |
| SIGLEC9 | CD80 | 0.78534149 | 7.40E-65 |
| SIGLEC9 | CD244 | 0.58063633 | 8.51E-29 |
| SIGLEC9 | TNFSF9 | 0.25624874 | 6.03E-06 |
| SIGLEC9 | CD70 | 0.42427701 | 1.03E-14 |
| SIGLEC9 | TNFSF14 | 0.47608015 | 1.33E-18 |
| SIGLEC9 | IDO1 | 0.48351449 | 3.23E-19 |
| SIGLEC9 | VTCN1 | -0.225207 | 7.45E-05 |
| SIGLEC9 | HAVCR2 | 0.89664721 | 7.03E-109 |
| SIGLEC9 | CD27 | 0.65752099 | 5.13E-39 |
| SIGLEC9 | TNFRSF14 | 0.35326551 | 2.30E-10 |
| SIGLEC9 | ICOSLG | 0.31212614 | 2.71E-08 |
| SIGLEC9 | CTLA4 | 0.69738519 | 1.34E-45 |
| SIGLEC9 | ICOS | 0.71640273 | 3.90E-49 |
| SIGLEC9 | CD200R1 | 0.53168123 | 1.40E-23 |
| SIGLEC9 | LAIR1 | 0.93046353 | 1.00E-133 |
| SIGLEC9 | KIR3DL1 | 0.38465257 | 3.70E-12 |
| SIGLEC9 | TMIGD2 | 0.39929671 | 4.60E-13 |
| SIGLEC9 | LGALS9 | 0.33626784 | 1.80E-09 |
| SIGLEC9 | CD28 | 0.6666055 | 1.99E-40 |
| SIGLEC9 | TIGIT | 0.67324396 | 1.73E-41 |
| SIGLEC9 | BTLA | 0.65562531 | 9.95E-39 |
| SIGLEC9 | TNFRSF4 | 0.65586989 | 9.14E-39 |
| SIGLEC9 | PDCD1 | 0.6667611 | 1.88E-40 |
| SIGLEC9 | IDO2 | 0.34822229 | 4.29E-10 |
| SIGLEC9 | PDCD1LG2 | 0.66478887 | 3.85E-40 |
| SIGLEC9 | TNFRSF25 | 0.19141194 | 0.0007945 |
