# Supplementary material for: The role of SIGLEC9 in immunosuppression and prognosis in cervical cancer
Source: Clinics (Sao Paulo). 2025 Dec 18;81:100849. doi: 10.1016/j.clinsp.2025.100849 (PMC12771336; doi:10.1016/j.clinsp.2025.100849)
Supplement: Supplementary file 3 [file mmc3.docx]

| ID | TCGA-MY-A5BF-11A-11R-A26T-07 | TCGA-HM-A3JJ-11A-12R-A21T-07 | TCGA-FU-A3EO-11A-13R-A213-07 | TCGA-ZJ-AAXB-01A-11R-A42T-07 | TCGA-IR-A3LH-01A-21R-A213-07 | TCGA-IR-A3LL-01A-11R-A213-07 | TCGA-VS-A8Q9-01A-12R-A37O-07 | TCGA-C5-A1MP-01A-11R-A14Y-07 | TCGA-C5-A902-01A-11R-A37O-07 |
| --- | --- | --- | --- | --- | --- | --- | --- | --- | --- |
| ST8SIA6 | 30 | 199 | 72 | 4 | 55 | 9 | 31 | 27 | 5 |
| ST6GALNAC5 | 28 | 64 | 51 | 39 | 778 | 35 | 76 | 190 | 13 |
| ST8SIA3 | 0 | 1 | 0 | 0 | 0 | 0 | 1 | 0 | 0 |
| ST8SIA5 | 5 | 40 | 11 | 23 | 6 | 14 | 53 | 2 | 3 |
| ST6GALNAC1 | 6318 | 380 | 311 | 396 | 88 | 4 | 246 | 196 | 14 |
| ST8SIA2 | 0 | 1 | 2 | 18 | 52 | 0 | 0 | 53 | 9 |
| ST6GAL2 | 125 | 484 | 122 | 75 | 270 | 22 | 10 | 244 | 27 |
| ST6GALNAC3 | 390 | 638 | 1144 | 27 | 107 | 32 | 24 | 44 | 14 |
| ST8SIA4 | 236 | 690 | 431 | 42 | 2830 | 1033 | 164 | 664 | 478 |
| ST3GAL3 | 29 | 69 | 43 | 17 | 85 | 44 | 81 | 50 | 68 |
| ST6GALNAC6 | 5 | 17 | 16 | 4 | 9 | 11 | 22 | 8 | 21 |
| ST6GALNAC2 | 31 | 81 | 56 | 10 | 487 | 159 | 842 | 2447 | 648 |
| ST3GAL4 | 2218 | 1535 | 1168 | 2895 | 1657 | 377 | 3652 | 963 | 2593 |
| ST3GAL6 | 430 | 583 | 292 | 555 | 382 | 351 | 102 | 232 | 196 |
| ST6GAL1 | 16749 | 4817 | 4880 | 1885 | 6069 | 1788 | 362 | 2672 | 496 |
| ST3GAL2 | 1073 | 4451 | 2812 | 246 | 2268 | 717 | 725 | 835 | 838 |
| ST3GAL1 | 3658 | 3324 | 3198 | 250 | 3111 | 3095 | 475 | 3499 | 1826 |
| ST6GALNAC4 | 1433 | 1428 | 1518 | 194 | 1299 | 417 | 1229 | 596 | 2413 |
| ST8SIA1 | 248 | 1097 | 391 | 338 | 278 | 108 | 51 | 153 | 43 |

| ID | TCGA-EK-A2IP-01A-11R-A180-07 | TCGA-HM-A6W2-01A-21R-A33Z-07 | TCGA-LP-A5U2-01A-11R-A28H-07 | TCGA-EK-A2RC-01A-11R-A18M-07 | TCGA-MA-AA3Y-01A-11R-A38B-07 | TCGA-C5-A1MK-01A-11R-A14Y-07 | TCGA-Q1-A73Q-01A-21R-A32P-07 | TCGA-VS-A8QA-01A-11R-A37O-07 | TCGA-C5-A1MQ-01A-11R-A14Y-07 |
| --- | --- | --- | --- | --- | --- | --- | --- | --- | --- |
| ST8SIA6 | 6 | 33 | 3 | 8 | 7 | 35 | 141 | 1 | 15 |
| ST6GALNAC5 | 73 | 42 | 13 | 66 | 46 | 223 | 95 | 2 | 78 |
| ST8SIA3 | 0 | 1 | 0 | 1 | 0 | 0 | 0 | 0 | 1 |
| ST8SIA5 | 6 | 160 | 10 | 6 | 5 | 7 | 1 | 55 | 3 |
| ST6GALNAC1 | 1448 | 3126 | 21 | 2201 | 21 | 500 | 172 | 1187 | 30 |
| ST8SIA2 | 2 | 3 | 3 | 11 | 6 | 69 | 5 | 7 | 15 |
| ST6GAL2 | 51 | 32 | 21 | 346 | 9 | 124 | 1758 | 0 | 24 |
| ST6GALNAC3 | 19 | 42 | 17 | 57 | 42 | 54 | 32 | 6 | 15 |
| ST8SIA4 | 152 | 257 | 190 | 503 | 583 | 293 | 201 | 157 | 444 |
| ST3GAL3 | 72 | 118 | 31 | 98 | 60 | 50 | 89 | 92 | 30 |
| ST6GALNAC6 | 5 | 7 | 37 | 7 | 8 | 3 | 3 | 31 | 3 |
| ST6GALNAC2 | 750 | 186 | 412 | 1062 | 463 | 392 | 1707 | 582 | 98 |
| ST3GAL4 | 1458 | 1971 | 675 | 719 | 1066 | 453 | 536 | 2687 | 2717 |
| ST3GAL6 | 1507 | 402 | 334 | 170 | 249 | 217 | 273 | 1707 | 99 |
| ST6GAL1 | 510 | 1424 | 586 | 3601 | 1536 | 564 | 701 | 16965 | 3346 |
| ST3GAL2 | 404 | 1718 | 460 | 615 | 714 | 715 | 647 | 1687 | 454 |
| ST3GAL1 | 2793 | 4814 | 360 | 4506 | 3359 | 5780 | 2829 | 353 | 1155 |
| ST6GALNAC4 | 646 | 954 | 3541 | 910 | 1246 | 783 | 446 | 2126 | 895 |
| ST8SIA1 | 28 | 33 | 32 | 89 | 60 | 136 | 80 | 22 | 127 |

| ID | TCGA-Q1-A73P-01A-11R-A32P-07 | TCGA-VS-A957-01A-11R-A42T-07 | TCGA-ZJ-AB0I-01A-11R-A42T-07 | TCGA-FU-A23K-01A-11R-A16R-07 | TCGA-C5-A907-01A-11R-A37O-07 | TCGA-IR-A3LI-01A-11R-A32Y-07 | TCGA-DR-A0ZM-01A-12R-A10U-07 | TCGA-FU-A3EO-01A-11R-A213-07 | TCGA-C5-A7X8-01A-11R-A36F-07 |
| --- | --- | --- | --- | --- | --- | --- | --- | --- | --- |
| ST8SIA6 | 9 | 1 | 1 | 16 | 29 | 70 | 5 | 3 | 52 |
| ST6GALNAC5 | 59 | 1 | 18 | 133 | 203 | 1 | 30 | 48 | 204 |
| ST8SIA3 | 0 | 1 | 0 | 0 | 1 | 1 | 0 | 0 | 0 |
| ST8SIA5 | 3 | 8 | 2 | 26 | 1 | 1 | 1 | 0 | 11 |
| ST6GALNAC1 | 8418 | 207 | 41 | 2623 | 449 | 3161 | 168 | 3162 | 912 |
| ST8SIA2 | 218 | 1 | 13 | 24 | 16 | 2 | 8 | 44 | 708 |
| ST6GAL2 | 74 | 2 | 17 | 210 | 8 | 3 | 23 | 24 | 862 |
| ST6GALNAC3 | 29 | 6 | 7 | 144 | 9 | 0 | 19 | 19 | 99 |
| ST8SIA4 | 318 | 50 | 101 | 510 | 211 | 13 | 685 | 93 | 501 |
| ST3GAL3 | 33 | 56 | 30 | 60 | 67 | 37 | 38 | 22 | 88 |
| ST6GALNAC6 | 31 | 7 | 5 | 44 | 6 | 26 | 6 | 30 | 33 |
| ST6GALNAC2 | 462 | 350 | 781 | 16 | 337 | 65 | 65 | 20 | 79 |
| ST3GAL4 | 2142 | 551 | 2362 | 791 | 4892 | 1774 | 347 | 2443 | 1767 |
| ST3GAL6 | 121 | 115 | 101 | 116 | 163 | 480 | 129 | 193 | 440 |
| ST6GAL1 | 17058 | 2254 | 518 | 3970 | 720 | 9264 | 534 | 39879 | 6575 |
| ST3GAL2 | 1126 | 222 | 392 | 875 | 392 | 641 | 358 | 364 | 1728 |
| ST3GAL1 | 2007 | 558 | 2111 | 592 | 6079 | 386 | 1436 | 2040 | 939 |
| ST6GALNAC4 | 3774 | 461 | 850 | 3793 | 399 | 1886 | 444 | 5379 | 3184 |
| ST8SIA1 | 71 | 13 | 13 | 110 | 24 | 56 | 104 | 9 | 78 |

| ID | TCGA-DS-A7WH-01A-22R-A352-07 | TCGA-EA-A5O9-01A-11R-A28H-07 | TCGA-DS-A1OA-01A-11R-A14Y-07 | TCGA-C5-A7CG-01A-11R-A32P-07 | TCGA-VS-A8EK-01A-12R-A37O-07 | TCGA-EK-A3GK-01A-11R-A213-07 | TCGA-C5-A1BN-01B-11R-A14Y-07 | TCGA-VS-A8EG-01A-11R-A36F-07 | TCGA-EA-A3QE-01A-21R-A21T-07 |
| --- | --- | --- | --- | --- | --- | --- | --- | --- | --- |
| ST8SIA6 | 122 | 3 | 13 | 67 | 21 | 9 | 15 | 116 | 22 |
| ST6GALNAC5 | 313 | 16 | 144 | 58 | 18 | 7 | 523 | 31 | 101 |
| ST8SIA3 | 0 | 1 | 0 | 0 | 0 | 0 | 0 | 1 | 1 |
| ST8SIA5 | 1 | 0 | 12 | 10 | 3 | 50 | 1 | 12 | 10 |
| ST6GALNAC1 | 505 | 568 | 1904 | 2265 | 639 | 909 | 3240 | 1399 | 3242 |
| ST8SIA2 | 45 | 1 | 2 | 0 | 5 | 0 | 6 | 2 | 0 |
| ST6GAL2 | 117 | 11 | 292 | 67 | 1 | 9 | 7 | 11 | 83 |
| ST6GALNAC3 | 83 | 11 | 31 | 80 | 8 | 18 | 26 | 32 | 63 |
| ST8SIA4 | 292 | 151 | 542 | 1446 | 281 | 271 | 103 | 301 | 1327 |
| ST3GAL3 | 120 | 37 | 63 | 108 | 59 | 42 | 72 | 113 | 56 |
| ST6GALNAC6 | 19 | 5 | 10 | 22 | 14 | 21 | 8 | 20 | 38 |
| ST6GALNAC2 | 46 | 1248 | 400 | 682 | 816 | 920 | 559 | 1952 | 1034 |
| ST3GAL4 | 3103 | 1001 | 1984 | 1383 | 380 | 27569 | 2545 | 1433 | 3173 |
| ST3GAL6 | 36 | 663 | 120 | 304 | 263 | 294 | 446 | 515 | 307 |
| ST6GAL1 | 15680 | 172 | 1598 | 2526 | 1195 | 22705 | 2192 | 6857 | 2397 |
| ST3GAL2 | 1352 | 291 | 750 | 985 | 859 | 623 | 647 | 662 | 1125 |
| ST3GAL1 | 11844 | 460 | 3369 | 1566 | 7159 | 4998 | 289 | 2318 | 8172 |
| ST6GALNAC4 | 2536 | 1076 | 1205 | 1644 | 1576 | 3171 | 1735 | 2497 | 2232 |
| ST8SIA1 | 27 | 263 | 189 | 483 | 26 | 41 | 34 | 46 | 364 |

| ID | TCGA-EA-A556-01A-11R-A26T-07 | TCGA-DS-A7WF-01A-11R-A352-07 | TCGA-EK-A2PK-01A-11R-A18M-07 | TCGA-UC-A7PF-01A-11R-A352-07 | TCGA-VS-A952-01A-11R-A38B-07 | TCGA-DS-A1OD-01A-11R-A14Y-07 | TCGA-Q1-A73R-01A-11R-A33Z-07 | TCGA-DS-A1O9-01A-11R-A14Y-07 | TCGA-EA-A5ZE-01A-11R-A28H-07 |
| --- | --- | --- | --- | --- | --- | --- | --- | --- | --- |
| ST8SIA6 | 40 | 1228 | 46 | 19 | 6 | 98 | 221 | 53 | 5 |
| ST6GALNAC5 | 524 | 92 | 27 | 144 | 5 | 87 | 26 | 400 | 67 |
| ST8SIA3 | 0 | 0 | 0 | 0 | 7 | 1 | 0 | 0 | 1 |
| ST8SIA5 | 56 | 6 | 1 | 19 | 2 | 2 | 10 | 2 | 6 |
| ST6GALNAC1 | 1275 | 1174 | 1843 | 343 | 3708 | 322 | 732 | 35 | 419 |
| ST8SIA2 | 130 | 18 | 4 | 6 | 3 | 3 | 1015 | 2 | 1 |
| ST6GAL2 | 163 | 35 | 48 | 198 | 23 | 369 | 89 | 89 | 189 |
| ST6GALNAC3 | 59 | 23 | 34 | 10 | 11 | 91 | 31 | 65 | 33 |
| ST8SIA4 | 311 | 772 | 868 | 524 | 162 | 1746 | 212 | 1308 | 649 |
| ST3GAL3 | 80 | 74 | 41 | 48 | 94 | 146 | 252 | 97 | 80 |
| ST6GALNAC6 | 10 | 13 | 10 | 4 | 81 | 25 | 16 | 10 | 3 |
| ST6GALNAC2 | 105 | 490 | 173 | 498 | 198 | 921 | 396 | 2022 | 422 |
| ST3GAL4 | 782 | 1881 | 2932 | 321 | 1092 | 2122 | 8153 | 1862 | 2620 |
| ST3GAL6 | 536 | 168 | 148 | 496 | 60 | 369 | 173 | 767 | 460 |
| ST6GAL1 | 1731 | 6716 | 3854 | 1827 | 5831 | 5087 | 7050 | 3255 | 15770 |
| ST3GAL2 | 3003 | 1625 | 789 | 930 | 824 | 2417 | 2381 | 2333 | 1022 |
| ST3GAL1 | 2026 | 440 | 4950 | 1089 | 1389 | 4386 | 466 | 6111 | 3024 |
| ST6GALNAC4 | 858 | 2294 | 1944 | 1506 | 3553 | 1870 | 1189 | 1377 | 1079 |
| ST8SIA1 | 581 | 43 | 61 | 45 | 45 | 1415 | 22 | 199 | 119 |

| ID | TCGA-LP-A4AX-01A-12R-A24H-07 | TCGA-FU-A3HZ-01A-11R-A213-07 | TCGA-VS-AA62-01A-11R-A42T-07 | TCGA-MY-A913-01A-11R-A37O-07 | TCGA-VS-A8QM-01A-11R-A37O-07 | TCGA-DS-A7WI-01A-12R-A352-07 | TCGA-C5-A7UE-01A-11R-A33Z-07 | TCGA-VS-A9UP-01A-11R-A42T-07 | TCGA-FU-A3NI-01A-11R-A21T-07 |
| --- | --- | --- | --- | --- | --- | --- | --- | --- | --- |
| ST8SIA6 | 6 | 175 | 22 | 4 | 60 | 4 | 79 | 101 | 8 |
| ST6GALNAC5 | 34 | 69 | 71 | 152 | 105 | 3 | 16 | 120 | 81 |
| ST8SIA3 | 0 | 2 | 0 | 0 | 0 | 0 | 3 | 0 | 0 |
| ST8SIA5 | 2 | 246 | 4 | 3 | 19 | 1 | 4 | 31 | 19 |
| ST6GALNAC1 | 8 | 1526 | 902 | 379 | 1893 | 60 | 478 | 1085 | 29 |
| ST8SIA2 | 5 | 160 | 36 | 2 | 2 | 3 | 0 | 104 | 4 |
| ST6GAL2 | 17 | 896 | 32 | 6 | 180 | 6 | 119 | 163 | 504 |
| ST6GALNAC3 | 75 | 33 | 31 | 29 | 101 | 4 | 1 | 17 | 48 |
| ST8SIA4 | 642 | 151 | 627 | 435 | 998 | 103 | 78 | 70 | 340 |
| ST3GAL3 | 52 | 35 | 67 | 72 | 68 | 82 | 47 | 153 | 45 |
| ST6GALNAC6 | 14 | 6 | 8 | 9 | 28 | 7 | 14 | 8 | 10 |
| ST6GALNAC2 | 116 | 195 | 635 | 637 | 800 | 126 | 238 | 654 | 2868 |
| ST3GAL4 | 1345 | 1079 | 1075 | 1804 | 1825 | 476 | 682 | 564 | 2213 |
| ST3GAL6 | 265 | 31 | 271 | 169 | 348 | 210 | 384 | 189 | 185 |
| ST6GAL1 | 2709 | 813 | 6529 | 4424 | 11621 | 1160 | 317 | 12318 | 1097 |
| ST3GAL2 | 1280 | 437 | 663 | 1118 | 1361 | 245 | 369 | 1419 | 821 |
| ST3GAL1 | 2043 | 395 | 4498 | 2290 | 3873 | 3313 | 2429 | 224 | 25880 |
| ST6GALNAC4 | 793 | 888 | 624 | 595 | 1373 | 1866 | 524 | 465 | 2065 |
| ST8SIA1 | 147 | 14 | 173 | 58 | 462 | 11 | 34 | 24 | 338 |

| ID | TCGA-VS-A8QC-01A-11R-A37O-07 | TCGA-ZJ-AAXJ-01A-11R-A42T-07 | TCGA-EA-A3HS-01A-11R-A213-07 | TCGA-VS-A94X-01A-11R-A38B-07 | TCGA-EX-A69M-01A-11R-A32P-07 | TCGA-EK-A2PM-01A-11R-A18M-07 | TCGA-ZJ-AAXI-01A-11R-A42T-07 | TCGA-EX-A8YF-01A-11R-A37O-07 | TCGA-VS-A8EH-01A-11R-A36F-07 |
| --- | --- | --- | --- | --- | --- | --- | --- | --- | --- |
| ST8SIA6 | 6 | 24 | 8 | 126 | 15 | 21 | 2 | 70 | 51 |
| ST6GALNAC5 | 115 | 41 | 138 | 46 | 36 | 162 | 2 | 62 | 23 |
| ST8SIA3 | 0 | 0 | 0 | 0 | 1 | 1 | 0 | 1 | 0 |
| ST8SIA5 | 103 | 8 | 10 | 9 | 1 | 1 | 8 | 16 | 47 |
| ST6GALNAC1 | 367 | 292 | 436 | 13 | 1319 | 15 | 278 | 867 | 1022 |
| ST8SIA2 | 22 | 0 | 6 | 10 | 0 | 9 | 1 | 3 | 2 |
| ST6GAL2 | 109 | 9 | 209 | 6 | 23 | 4915 | 13 | 42 | 15 |
| ST6GALNAC3 | 14 | 5 | 29 | 10 | 56 | 40 | 6 | 43 | 7 |
| ST8SIA4 | 208 | 132 | 326 | 157 | 596 | 203 | 105 | 497 | 136 |
| ST3GAL3 | 68 | 92 | 154 | 34 | 158 | 40 | 59 | 38 | 158 |
| ST6GALNAC6 | 12 | 2 | 11 | 11 | 12 | 10 | 14 | 12 | 12 |
| ST6GALNAC2 | 726 | 98 | 1690 | 401 | 477 | 541 | 112 | 492 | 1015 |
| ST3GAL4 | 724 | 764 | 812 | 1974 | 2053 | 376 | 87 | 7989 | 855 |
| ST3GAL6 | 265 | 159 | 365 | 113 | 142 | 148 | 437 | 252 | 94 |
| ST6GAL1 | 760 | 1405 | 1157 | 2771 | 3766 | 682 | 1579 | 6942 | 1854 |
| ST3GAL2 | 824 | 192 | 1379 | 436 | 341 | 353 | 441 | 401 | 678 |
| ST3GAL1 | 2472 | 2111 | 1023 | 2969 | 4342 | 1442 | 952 | 9988 | 3390 |
| ST6GALNAC4 | 1121 | 142 | 1613 | 1492 | 1987 | 999 | 280 | 1665 | 2339 |
| ST8SIA1 | 499 | 82 | 269 | 22 | 86 | 62 | 54 | 75 | 46 |

| ID | TCGA-EK-A2R8-01A-21R-A18M-07 | TCGA-EK-A2RM-01A-21R-A18M-07 | TCGA-C5-A1BM-01A-11R-A13Y-07 | TCGA-DS-A3LQ-01A-21R-A21T-07 | TCGA-C5-A2M1-01A-11R-A18M-07 | TCGA-DS-A1OC-01A-11R-A14Y-07 | TCGA-VS-A958-01A-11R-A42T-07 | TCGA-EA-A410-01A-11R-A32Y-07 | TCGA-HM-A3JJ-01A-21R-A21T-07 |
| --- | --- | --- | --- | --- | --- | --- | --- | --- | --- |
| ST8SIA6 | 88 | 169 | 15 | 32 | 10 | 6 | 7 | 40 | 251 |
| ST6GALNAC5 | 65 | 554 | 36 | 287 | 41 | 83 | 9 | 39 | 454 |
| ST8SIA3 | 0 | 0 | 0 | 0 | 1 | 0 | 0 | 0 | 0 |
| ST8SIA5 | 1 | 4 | 9 | 11 | 13 | 10 | 2 | 8 | 24 |
| ST6GALNAC1 | 1767 | 7561 | 175 | 536 | 14112 | 678 | 27 | 7 | 1221 |
| ST8SIA2 | 2 | 80 | 41 | 81 | 124 | 3 | 3 | 3908 | 2 |
| ST6GAL2 | 49 | 234 | 46 | 198 | 67 | 31 | 0 | 1257 | 520 |
| ST6GALNAC3 | 22 | 8 | 42 | 56 | 54 | 54 | 27 | 48 | 58 |
| ST8SIA4 | 198 | 147 | 1040 | 648 | 927 | 400 | 468 | 333 | 540 |
| ST3GAL3 | 27 | 70 | 66 | 49 | 54 | 42 | 36 | 58 | 50 |
| ST6GALNAC6 | 6 | 3 | 6 | 1 | 47 | 6 | 10 | 2 | 21 |
| ST6GALNAC2 | 274 | 59 | 525 | 859 | 938 | 298 | 664 | 38 | 979 |
| ST3GAL4 | 6162 | 7763 | 532 | 1500 | 4893 | 661 | 193 | 1037 | 1840 |
| ST3GAL6 | 420 | 261 | 719 | 132 | 1069 | 183 | 197 | 82 | 377 |
| ST6GAL1 | 2324 | 10961 | 2891 | 1098 | 18916 | 2097 | 1264 | 554 | 6191 |
| ST3GAL2 | 952 | 934 | 570 | 779 | 1156 | 500 | 532 | 915 | 1852 |
| ST3GAL1 | 5344 | 2317 | 9144 | 3018 | 4984 | 9121 | 4551 | 754 | 5938 |
| ST6GALNAC4 | 1237 | 1494 | 514 | 1404 | 8104 | 1361 | 730 | 420 | 2571 |
| ST8SIA1 | 14 | 17 | 82 | 159 | 153 | 63 | 74 | 41 | 263 |

| ID | TCGA-EK-A3GN-01A-11R-A213-07 | TCGA-HG-A2PA-01A-11R-A213-07 | TCGA-Q1-A73O-01A-11R-A32P-07 | TCGA-VS-A9UC-01A-11R-A42T-07 | TCGA-Q1-A6DT-01A-11R-A32P-07 | TCGA-VS-A94W-01A-12R-A37O-07 | TCGA-LP-A7HU-01A-11R-A33Z-07 | TCGA-EK-A2H0-01A-11R-A180-07 | TCGA-FU-A3YQ-01A-11R-A22U-07 |
| --- | --- | --- | --- | --- | --- | --- | --- | --- | --- |
| ST8SIA6 | 22 | 323 | 33 | 15 | 2 | 7 | 1 | 82 | 50 |
| ST6GALNAC5 | 38 | 119 | 58 | 44 | 5 | 18 | 71 | 139 | 182 |
| ST8SIA3 | 1 | 1 | 0 | 0 | 1 | 1 | 0 | 0 | 0 |
| ST8SIA5 | 19 | 6 | 4 | 1 | 0 | 10 | 36 | 12 | 3 |
| ST6GALNAC1 | 333 | 10368 | 886 | 994 | 4 | 476 | 54 | 441 | 639 |
| ST8SIA2 | 2 | 109 | 25 | 12 | 6 | 11 | 28 | 4 | 9 |
| ST6GAL2 | 27 | 80 | 35 | 115 | 3 | 58 | 36 | 7 | 311 |
| ST6GALNAC3 | 9 | 47 | 27 | 15 | 14 | 112 | 11 | 2 | 89 |
| ST8SIA4 | 54 | 1168 | 390 | 369 | 115 | 712 | 279 | 171 | 682 |
| ST3GAL3 | 197 | 142 | 58 | 88 | 72 | 126 | 77 | 97 | 57 |
| ST6GALNAC6 | 12 | 16 | 7 | 2 | 13 | 45 | 30 | 12 | 7 |
| ST6GALNAC2 | 299 | 1139 | 496 | 730 | 187 | 508 | 29 | 826 | 561 |
| ST3GAL4 | 4199 | 3387 | 682 | 5738 | 701 | 561 | 598 | 1060 | 2194 |
| ST3GAL6 | 41 | 327 | 93 | 500 | 350 | 538 | 175 | 227 | 345 |
| ST6GAL1 | 4140 | 7900 | 3376 | 645 | 1311 | 2133 | 3251 | 197 | 3471 |
| ST3GAL2 | 1315 | 1049 | 713 | 776 | 502 | 1900 | 861 | 615 | 1206 |
| ST3GAL1 | 1034 | 6743 | 4197 | 3972 | 6068 | 1811 | 538 | 1699 | 13971 |
| ST6GALNAC4 | 1562 | 1991 | 712 | 704 | 1613 | 1704 | 2265 | 853 | 1667 |
| ST8SIA1 | 16 | 158 | 159 | 226 | 12 | 868 | 154 | 40 | 174 |

| ID | TCGA-EA-A6QX-01A-12R-A33Z-07 | TCGA-VS-A8Q8-01A-11R-A37O-07 | TCGA-EK-A2RA-01A-11R-A18M-07 | TCGA-FU-A23L-01A-11R-A16R-07 | TCGA-C5-A7UC-01A-11R-A352-07 | TCGA-C5-A1BQ-01C-11R-A213-07 | TCGA-WL-A834-01A-11R-A352-07 | TCGA-C5-A2LV-01A-11R-A18M-07 | TCGA-VS-A9UY-01A-11R-A42T-07 |
| --- | --- | --- | --- | --- | --- | --- | --- | --- | --- |
| ST8SIA6 | 14 | 57 | 222 | 28 | 17 | 5 | 5 | 14 | 9 |
| ST6GALNAC5 | 10 | 13 | 10 | 226 | 89 | 87 | 42 | 134 | 10 |
| ST8SIA3 | 6 | 0 | 4 | 0 | 0 | 0 | 3 | 0 | 2 |
| ST8SIA5 | 2 | 0 | 1 | 20 | 3 | 2 | 1 | 16 | 6 |
| ST6GALNAC1 | 555 | 319 | 1677 | 1099 | 1111 | 14 | 3446 | 5 | 109 |
| ST8SIA2 | 3 | 1 | 0 | 2 | 17 | 9 | 1 | 8 | 0 |
| ST6GAL2 | 43 | 28 | 28 | 28 | 34 | 13 | 71 | 6 | 16 |
| ST6GALNAC3 | 18 | 11 | 26 | 85 | 11 | 48 | 20 | 29 | 17 |
| ST8SIA4 | 304 | 318 | 571 | 727 | 60 | 536 | 131 | 500 | 320 |
| ST3GAL3 | 30 | 22 | 145 | 134 | 101 | 79 | 54 | 27 | 71 |
| ST6GALNAC6 | 15 | 14 | 9 | 9 | 11 | 7 | 6 | 6 | 4 |
| ST6GALNAC2 | 713 | 275 | 1177 | 1739 | 859 | 305 | 764 | 260 | 871 |
| ST3GAL4 | 1343 | 1252 | 930 | 2030 | 1614 | 326 | 581 | 1958 | 801 |
| ST3GAL6 | 96 | 409 | 308 | 469 | 559 | 282 | 439 | 266 | 111 |
| ST6GAL1 | 9311 | 578 | 1984 | 7163 | 1897 | 640 | 4247 | 1203 | 591 |
| ST3GAL2 | 757 | 344 | 744 | 1345 | 445 | 553 | 483 | 977 | 351 |
| ST3GAL1 | 3380 | 1811 | 3707 | 1546 | 3612 | 4581 | 6695 | 1104 | 7158 |
| ST6GALNAC4 | 2947 | 988 | 1286 | 1767 | 1879 | 1015 | 1521 | 1160 | 1357 |
| ST8SIA1 | 55 | 93 | 274 | 178 | 36 | 56 | 206 | 72 | 68 |

| ID | TCGA-C5-A1MN-01A-11R-A14Y-07 | TCGA-C5-A2LS-01A-22R-A22U-07 | TCGA-C5-A7X3-01A-11R-A352-07 | TCGA-EA-A5FO-01A-21R-A28H-07 | TCGA-VS-A9V5-01A-11R-A42T-07 | TCGA-FU-A40J-01A-11R-A24H-07 | TCGA-ZJ-AB0H-01A-11R-A42T-07 | TCGA-ZJ-A8QQ-01A-11R-A37O-07 | TCGA-EA-A78R-01A-11R-A32P-07 |
| --- | --- | --- | --- | --- | --- | --- | --- | --- | --- |
| ST8SIA6 | 65 | 35 | 4 | 0 | 8 | 50 | 139 | 34 | 65 |
| ST6GALNAC5 | 65 | 7 | 136 | 2 | 19 | 121 | 137 | 97 | 79 |
| ST8SIA3 | 0 | 0 | 0 | 0 | 13 | 786 | 0 | 0 | 0 |
| ST8SIA5 | 2 | 7 | 5 | 7 | 6 | 10 | 0 | 8 | 1 |
| ST6GALNAC1 | 111 | 7706 | 61 | 2392 | 5362 | 2976 | 289 | 309 | 1360 |
| ST8SIA2 | 0 | 431 | 21 | 0 | 16 | 8 | 10 | 3 | 8 |
| ST6GAL2 | 1 | 10 | 28 | 3 | 32 | 118 | 42 | 10 | 49 |
| ST6GALNAC3 | 18 | 16 | 8 | 12 | 39 | 31 | 25 | 16 | 17 |
| ST8SIA4 | 87 | 182 | 351 | 50 | 175 | 679 | 430 | 112 | 188 |
| ST3GAL3 | 78 | 55 | 109 | 86 | 50 | 170 | 75 | 48 | 50 |
| ST6GALNAC6 | 3 | 39 | 8 | 19 | 91 | 52 | 8 | 9 | 3 |
| ST6GALNAC2 | 330 | 329 | 266 | 1116 | 535 | 497 | 490 | 970 | 122 |
| ST3GAL4 | 3740 | 1853 | 2733 | 1186 | 1511 | 4338 | 694 | 764 | 428 |
| ST3GAL6 | 258 | 654 | 165 | 171 | 405 | 284 | 95 | 148 | 271 |
| ST6GAL1 | 171 | 16017 | 5154 | 6305 | 33377 | 71632 | 3357 | 304 | 1834 |
| ST3GAL2 | 1321 | 258 | 984 | 361 | 416 | 1138 | 690 | 610 | 420 |
| ST3GAL1 | 745 | 2003 | 2346 | 990 | 2595 | 1984 | 863 | 1816 | 1074 |
| ST6GALNAC4 | 1407 | 2859 | 1382 | 1389 | 5788 | 4686 | 1191 | 1107 | 1221 |
| ST8SIA1 | 27 | 92 | 65 | 32 | 26 | 134 | 196 | 27 | 98 |

| ID | TCGA-EK-A2RO-01A-11R-A18M-07 | TCGA-C5-A2M2-01A-21R-A18M-07 | TCGA-C5-A7UH-01A-11R-A352-07 | TCGA-VS-A9UQ-01A-21R-A42T-07 | TCGA-EX-A69L-01A-11R-A32P-07 | TCGA-VS-A9U5-01A-11R-A42T-07 | TCGA-EA-A3Y4-01A-51R-A24H-07 | TCGA-BI-A20A-01A-11R-A14Y-07 | TCGA-C5-A8YQ-01A-11R-A37O-07 |
| --- | --- | --- | --- | --- | --- | --- | --- | --- | --- |
| ST8SIA6 | 24 | 1 | 5 | 7 | 37 | 3 | 18 | 12 | 1 |
| ST6GALNAC5 | 307 | 23 | 50 | 158 | 45 | 38 | 64 | 13 | 190 |
| ST8SIA3 | 0 | 1 | 0 | 0 | 0 | 0 | 0 | 0 | 0 |
| ST8SIA5 | 12 | 3 | 15 | 5 | 2 | 1 | 1 | 3 | 8 |
| ST6GALNAC1 | 1150 | 10527 | 840 | 6905 | 379 | 3329 | 251 | 2211 | 827 |
| ST8SIA2 | 78 | 18 | 7 | 28 | 13 | 0 | 7 | 6 | 0 |
| ST6GAL2 | 63 | 11 | 28 | 270 | 81 | 14 | 92 | 151 | 38 |
| ST6GALNAC3 | 18 | 21 | 32 | 34 | 43 | 16 | 31 | 51 | 6 |
| ST8SIA4 | 92 | 135 | 309 | 187 | 818 | 272 | 743 | 461 | 145 |
| ST3GAL3 | 57 | 38 | 118 | 119 | 42 | 94 | 90 | 186 | 100 |
| ST6GALNAC6 | 4 | 43 | 7 | 25 | 8 | 10 | 16 | 8 | 10 |
| ST6GALNAC2 | 435 | 273 | 529 | 1664 | 928 | 430 | 109 | 1454 | 1675 |
| ST3GAL4 | 596 | 358 | 432 | 649 | 495 | 199 | 8468 | 738 | 1001 |
| ST3GAL6 | 466 | 331 | 126 | 421 | 163 | 33 | 116 | 204 | 312 |
| ST6GAL1 | 3938 | 21507 | 805 | 26048 | 2316 | 2906 | 2674 | 3371 | 447 |
| ST3GAL2 | 272 | 785 | 738 | 734 | 938 | 386 | 675 | 924 | 1144 |
| ST3GAL1 | 1144 | 3089 | 2012 | 1870 | 2113 | 378 | 5282 | 1736 | 965 |
| ST6GALNAC4 | 1109 | 6338 | 1237 | 2341 | 1142 | 521 | 877 | 2025 | 1478 |
| ST8SIA1 | 52 | 58 | 73 | 103 | 163 | 84 | 199 | 151 | 1619 |

| ID | TCGA-EA-A3HR-01A-11R-A213-07 | TCGA-FU-A2QG-01A-11R-A18M-07 | TCGA-C5-A1BI-01B-11R-A13Y-07 | TCGA-EA-A3HT-01A-61R-A21T-07 | TCGA-IR-A3LF-01A-21R-A22U-07 | TCGA-BI-A0VS-01A-11R-A10U-07 | TCGA-EX-A449-01A-11R-A32Y-07 | TCGA-VS-A9UV-01A-11R-A42T-07 | TCGA-LP-A5U3-01A-11R-A28H-07 |
| --- | --- | --- | --- | --- | --- | --- | --- | --- | --- |
| ST8SIA6 | 15 | 92 | 20 | 106 | 21 | 5 | 297 | 6 | 114 |
| ST6GALNAC5 | 180 | 31 | 289 | 1322 | 79 | 293 | 4 | 41 | 5 |
| ST8SIA3 | 0 | 0 | 1 | 0 | 14 | 0 | 0 | 0 | 0 |
| ST8SIA5 | 20 | 6 | 7 | 8 | 23 | 50 | 6 | 3 | 0 |
| ST6GALNAC1 | 2324 | 1726 | 4203 | 1979 | 4040 | 447 | 11065 | 634 | 2642 |
| ST8SIA2 | 7 | 5 | 6 | 78 | 110 | 19 | 114 | 3 | 2 |
| ST6GAL2 | 418 | 8 | 259 | 262 | 66 | 165 | 34 | 0 | 14 |
| ST6GALNAC3 | 53 | 29 | 49 | 54 | 71 | 38 | 8 | 17 | 15 |
| ST8SIA4 | 321 | 272 | 920 | 502 | 478 | 425 | 90 | 98 | 370 |
| ST3GAL3 | 86 | 95 | 94 | 95 | 125 | 100 | 80 | 94 | 26 |
| ST6GALNAC6 | 3 | 8 | 7 | 16 | 34 | 2 | 89 | 11 | 2 |
| ST6GALNAC2 | 3304 | 1146 | 1067 | 1131 | 425 | 1016 | 199 | 299 | 817 |
| ST3GAL4 | 1149 | 3611 | 2716 | 976 | 2210 | 1780 | 1250 | 418 | 898 |
| ST3GAL6 | 456 | 449 | 234 | 1133 | 1532 | 386 | 455 | 33 | 140 |
| ST6GAL1 | 1932 | 3396 | 2598 | 8471 | 4038 | 2800 | 5022 | 882 | 1025 |
| ST3GAL2 | 905 | 587 | 1906 | 1073 | 1454 | 2312 | 765 | 451 | 432 |
| ST3GAL1 | 6273 | 2170 | 6938 | 11482 | 4827 | 9858 | 754 | 367 | 2047 |
| ST6GALNAC4 | 651 | 2727 | 1069 | 1340 | 2172 | 1113 | 9462 | 1018 | 690 |
| ST8SIA1 | 266 | 30 | 871 | 81 | 171 | 434 | 21 | 29 | 72 |

| ID | TCGA-UC-A7PG-06A-11R-A42S-07 | TCGA-2W-A8YY-01A-11R-A37O-07 | TCGA-C5-A2LT-01A-11R-A18M-07 | TCGA-C5-A2LX-01A-11R-A18M-07 | TCGA-ZJ-AAXU-01A-11R-A42T-07 | TCGA-IR-A3LC-01A-11R-A213-07 | TCGA-VS-A953-01A-11R-A38B-07 | TCGA-EK-A2RL-01A-11R-A18M-07 | TCGA-VS-A9UH-01A-11R-A42T-07 |
| --- | --- | --- | --- | --- | --- | --- | --- | --- | --- |
| ST8SIA6 | 8 | 3 | 27 | 18 | 21 | 17 | 1 | 3 | 0 |
| ST6GALNAC5 | 84 | 47 | 275 | 41 | 37 | 52 | 3 | 91 | 121 |
| ST8SIA3 | 0 | 4 | 2 | 0 | 0 | 0 | 0 | 0 | 1 |
| ST8SIA5 | 8 | 35 | 23 | 6 | 60 | 2 | 13 | 17 | 24 |
| ST6GALNAC1 | 766 | 444 | 51 | 613 | 888 | 2682 | 16 | 8582 | 16 |
| ST8SIA2 | 2 | 2 | 29 | 3 | 6 | 3 | 1 | 11 | 7 |
| ST6GAL2 | 100 | 18 | 58 | 60 | 9 | 114 | 2 | 115 | 22 |
| ST6GALNAC3 | 26 | 45 | 58 | 47 | 17 | 24 | 8 | 23 | 34 |
| ST8SIA4 | 485 | 270 | 404 | 730 | 465 | 271 | 202 | 223 | 532 |
| ST3GAL3 | 164 | 84 | 57 | 76 | 67 | 60 | 100 | 62 | 73 |
| ST6GALNAC6 | 12 | 9 | 9 | 12 | 33 | 5 | 11 | 23 | 12 |
| ST6GALNAC2 | 924 | 135 | 1001 | 226 | 363 | 1558 | 459 | 465 | 270 |
| ST3GAL4 | 212 | 1762 | 2460 | 561 | 1081 | 1089 | 728 | 3052 | 793 |
| ST3GAL6 | 541 | 213 | 1129 | 199 | 115 | 51 | 394 | 1126 | 295 |
| ST6GAL1 | 1053 | 2361 | 3040 | 1660 | 3724 | 778 | 211 | 34841 | 1859 |
| ST3GAL2 | 731 | 392 | 1258 | 932 | 1358 | 757 | 1297 | 535 | 1270 |
| ST3GAL1 | 4824 | 1806 | 7076 | 713 | 1538 | 4664 | 5945 | 1755 | 2546 |
| ST6GALNAC4 | 964 | 1461 | 2151 | 1763 | 2156 | 2037 | 911 | 4386 | 1330 |
| ST8SIA1 | 172 | 45 | 102 | 846 | 424 | 24 | 20 | 45 | 102 |

| ID | TCGA-VS-A9UU-01A-11R-A42T-07 | TCGA-C5-A1BK-01B-11R-A13Y-07 | TCGA-EK-A2H1-01A-11R-A180-07 | TCGA-EX-A1H6-01B-11R-A22U-07 | TCGA-ZJ-AAX8-01A-11R-A42T-07 | TCGA-VS-A9UR-01A-11R-A42T-07 | TCGA-C5-A1M9-01A-11R-A13Y-07 | TCGA-VS-A950-01A-11R-A42T-07 | TCGA-DS-A1OB-01A-11R-A14Y-07 |
| --- | --- | --- | --- | --- | --- | --- | --- | --- | --- |
| ST8SIA6 | 4 | 10 | 17 | 13 | 29 | 43 | 2 | 0 | 3 |
| ST6GALNAC5 | 6 | 187 | 183 | 95 | 332 | 10 | 80 | 39 | 194 |
| ST8SIA3 | 0 | 0 | 0 | 0 | 0 | 0 | 1 | 0 | 0 |
| ST8SIA5 | 1 | 3 | 1 | 9 | 11 | 75 | 3 | 2 | 32 |
| ST6GALNAC1 | 621 | 1835 | 217 | 2670 | 104 | 1995 | 5109 | 81 | 45 |
| ST8SIA2 | 2 | 1 | 40 | 5 | 31 | 75 | 13 | 16 | 8 |
| ST6GAL2 | 0 | 62 | 21 | 110 | 169 | 1 | 12 | 34 | 38 |
| ST6GALNAC3 | 4 | 33 | 39 | 45 | 26 | 6 | 31 | 20 | 29 |
| ST8SIA4 | 88 | 1008 | 446 | 192 | 406 | 75 | 325 | 310 | 355 |
| ST3GAL3 | 73 | 49 | 55 | 59 | 186 | 82 | 108 | 81 | 70 |
| ST6GALNAC6 | 22 | 21 | 10 | 30 | 14 | 32 | 6 | 16 | 5 |
| ST6GALNAC2 | 145 | 725 | 183 | 87 | 627 | 306 | 373 | 363 | 856 |
| ST3GAL4 | 154 | 1228 | 1408 | 2246 | 666 | 1601 | 7947 | 2038 | 1039 |
| ST3GAL6 | 106 | 173 | 446 | 426 | 660 | 219 | 119 | 66 | 306 |
| ST6GAL1 | 1521 | 1918 | 1477 | 3312 | 8888 | 23402 | 4696 | 1323 | 462 |
| ST3GAL2 | 387 | 721 | 1369 | 583 | 973 | 739 | 713 | 813 | 324 |
| ST3GAL1 | 3567 | 1643 | 4044 | 3672 | 4994 | 4204 | 7038 | 1650 | 4475 |
| ST6GALNAC4 | 1004 | 1202 | 2058 | 2726 | 1132 | 1284 | 933 | 1141 | 287 |
| ST8SIA1 | 19 | 346 | 77 | 81 | 386 | 22 | 37 | 48 | 79 |

| ID | TCGA-EK-A2R9-01A-11R-A18M-07 | TCGA-FU-A3TQ-01A-11R-A22U-07 | TCGA-C5-A8XH-01A-11R-A37O-07 | TCGA-VS-A9UD-01A-11R-A42T-07 | TCGA-FU-A3TX-01A-11R-A22U-07 | TCGA-C5-A7CH-01A-11R-A33Z-07 | TCGA-Q1-A73S-01A-11R-A33Z-07 | TCGA-EK-A2R7-01A-11R-A18M-07 | TCGA-HM-A3JK-01A-11R-A32Y-07 |
| --- | --- | --- | --- | --- | --- | --- | --- | --- | --- |
| ST8SIA6 | 6 | 468 | 6 | 2 | 16 | 30 | 527 | 8 | 51 |
| ST6GALNAC5 | 200 | 146 | 28 | 7 | 9 | 34 | 66 | 64 | 486 |
| ST8SIA3 | 0 | 0 | 1 | 0 | 0 | 0 | 0 | 0 | 0 |
| ST8SIA5 | 35 | 2 | 8 | 8 | 17 | 0 | 2 | 17 | 5 |
| ST6GALNAC1 | 50 | 12386 | 201 | 110 | 78 | 278 | 3154 | 10295 | 2672 |
| ST8SIA2 | 346 | 16 | 2 | 2 | 11 | 12 | 22 | 56 | 61 |
| ST6GAL2 | 140 | 64 | 76 | 0 | 395 | 26 | 35 | 48 | 432 |
| ST6GALNAC3 | 47 | 32 | 20 | 17 | 113 | 17 | 3 | 123 | 46 |
| ST8SIA4 | 557 | 477 | 334 | 303 | 440 | 111 | 97 | 509 | 233 |
| ST3GAL3 | 93 | 118 | 91 | 24 | 132 | 70 | 89 | 226 | 59 |
| ST6GALNAC6 | 4 | 8 | 6 | 9 | 25 | 9 | 0 | 36 | 3 |
| ST6GALNAC2 | 506 | 3337 | 1932 | 215 | 393 | 444 | 555 | 720 | 867 |
| ST3GAL4 | 1114 | 2877 | 977 | 289 | 5254 | 1106 | 3353 | 5321 | 996 |
| ST3GAL6 | 729 | 237 | 467 | 152 | 2150 | 257 | 122 | 182 | 328 |
| ST6GAL1 | 3042 | 2264 | 1228 | 1084 | 3538 | 1012 | 736 | 21517 | 5914 |
| ST3GAL2 | 1261 | 621 | 567 | 482 | 1465 | 642 | 541 | 1002 | 1094 |
| ST3GAL1 | 1743 | 4359 | 1943 | 968 | 2330 | 1633 | 2539 | 2100 | 2138 |
| ST6GALNAC4 | 1572 | 1412 | 1353 | 634 | 3279 | 1499 | 812 | 3261 | 1250 |
| ST8SIA1 | 175 | 132 | 126 | 60 | 190 | 40 | 20 | 163 | 114 |

| ID | TCGA-FU-A3HY-01A-11R-A21T-07 | TCGA-C5-A1MJ-01A-11R-A14Y-07 | TCGA-DG-A2KM-01A-11R-A180-07 | TCGA-JW-AAVH-01A-11R-A38B-07 | TCGA-C5-A1BJ-01A-11R-A13Y-07 | TCGA-JX-A3PZ-01A-11R-A32Y-07 | TCGA-VS-A8QH-01A-11R-A37O-07 | TCGA-C5-A2LZ-01A-11R-A213-07 | TCGA-JW-A5VJ-01A-11R-A28H-07 |
| --- | --- | --- | --- | --- | --- | --- | --- | --- | --- |
| ST8SIA6 | 4 | 23 | 93 | 6 | 72 | 10 | 286 | 23 | 9 |
| ST6GALNAC5 | 53 | 656 | 230 | 35 | 606 | 269 | 14 | 44 | 31 |
| ST8SIA3 | 0 | 6 | 1 | 0 | 0 | 0 | 3 | 0 | 0 |
| ST8SIA5 | 7 | 4 | 5 | 8 | 10 | 1 | 3 | 4 | 14 |
| ST6GALNAC1 | 3076 | 1743 | 5190 | 3815 | 3619 | 277 | 8225 | 1029 | 411 |
| ST8SIA2 | 1 | 271 | 3 | 1 | 14 | 28 | 275 | 6 | 113 |
| ST6GAL2 | 48 | 244 | 130 | 28 | 365 | 64 | 36 | 14 | 19 |
| ST6GALNAC3 | 19 | 169 | 265 | 11 | 56 | 17 | 21 | 19 | 16 |
| ST8SIA4 | 101 | 785 | 2494 | 80 | 861 | 336 | 54 | 325 | 160 |
| ST3GAL3 | 66 | 121 | 56 | 230 | 156 | 42 | 10 | 98 | 229 |
| ST6GALNAC6 | 3 | 23 | 26 | 12 | 17 | 1 | 16 | 15 | 15 |
| ST6GALNAC2 | 386 | 168 | 1130 | 675 | 1202 | 402 | 42 | 863 | 635 |
| ST3GAL4 | 3567 | 2682 | 1722 | 319 | 1380 | 3208 | 5969 | 406 | 1762 |
| ST3GAL6 | 1021 | 356 | 726 | 373 | 320 | 185 | 38 | 1047 | 417 |
| ST6GAL1 | 2704 | 10273 | 14707 | 1019 | 10058 | 672 | 3935 | 1946 | 506 |
| ST3GAL2 | 640 | 1860 | 1930 | 337 | 2041 | 748 | 648 | 661 | 733 |
| ST3GAL1 | 3488 | 3801 | 8183 | 517 | 3613 | 2091 | 792 | 5430 | 5271 |
| ST6GALNAC4 | 1067 | 3373 | 1497 | 818 | 3299 | 1245 | 1626 | 1444 | 1182 |
| ST8SIA1 | 125 | 67 | 663 | 30 | 299 | 13 | 12 | 61 | 692 |

| ID | TCGA-VS-A9UZ-01A-11R-A42T-07 | TCGA-IR-A3L7-01A-21R-A213-07 | TCGA-EK-A2GZ-01A-11R-A180-07 | TCGA-JW-A5VK-01A-11R-A28H-07 | TCGA-EK-A2RJ-01A-11R-A18M-07 | TCGA-ZJ-AAXT-01A-11R-A42T-07 | TCGA-MA-AA3Z-01A-11R-A38B-07 | TCGA-C5-A1BL-01A-11R-A13Y-07 | TCGA-Q1-A5R3-01A-11R-A28H-07 |
| --- | --- | --- | --- | --- | --- | --- | --- | --- | --- |
| ST8SIA6 | 1 | 13 | 215 | 2 | 27 | 4 | 3 | 6 | 25 |
| ST6GALNAC5 | 16 | 37 | 34 | 4 | 28 | 22 | 61 | 65 | 111 |
| ST8SIA3 | 0 | 2 | 0 | 0 | 0 | 0 | 0 | 0 | 2 |
| ST8SIA5 | 14 | 10 | 3 | 5 | 3 | 2 | 2 | 3 | 0 |
| ST6GALNAC1 | 801 | 10817 | 326 | 312 | 41 | 105 | 1285 | 695 | 1294 |
| ST8SIA2 | 64 | 4 | 36 | 10 | 6 | 8 | 0 | 0 | 0 |
| ST6GAL2 | 66 | 177 | 19 | 36 | 16 | 7 | 10 | 25 | 13 |
| ST6GALNAC3 | 35 | 43 | 11 | 9 | 32 | 8 | 14 | 17 | 4 |
| ST8SIA4 | 205 | 1262 | 217 | 71 | 669 | 393 | 294 | 371 | 254 |
| ST3GAL3 | 70 | 130 | 47 | 123 | 153 | 76 | 36 | 87 | 42 |
| ST6GALNAC6 | 34 | 30 | 7 | 16 | 4 | 5 | 11 | 9 | 0 |
| ST6GALNAC2 | 124 | 2359 | 1465 | 340 | 304 | 335 | 926 | 398 | 25 |
| ST3GAL4 | 4336 | 4594 | 1078 | 585 | 9842 | 1338 | 2193 | 285 | 2113 |
| ST3GAL6 | 256 | 442 | 742 | 206 | 101 | 321 | 95 | 120 | 423 |
| ST6GAL1 | 39963 | 17175 | 5362 | 440 | 2094 | 464 | 2745 | 4869 | 6151 |
| ST3GAL2 | 1249 | 935 | 613 | 572 | 495 | 860 | 617 | 401 | 396 |
| ST3GAL1 | 5784 | 3273 | 7188 | 2718 | 2309 | 2197 | 4270 | 3579 | 5138 |
| ST6GALNAC4 | 3906 | 2832 | 1222 | 1492 | 372 | 396 | 1412 | 733 | 2762 |
| ST8SIA1 | 53 | 42 | 194 | 129 | 66 | 239 | 55 | 33 | 80 |

| ID | TCGA-C5-A7CM-01A-11R-A33Z-07 | TCGA-ZJ-A8QR-01A-11R-A37O-07 | TCGA-C5-A8XJ-01A-11R-A37O-07 | TCGA-VS-A9UL-01A-11R-A42T-07 | TCGA-EA-A3HQ-01A-11R-A213-07 | TCGA-C5-A1BF-01B-11R-A13Y-07 | TCGA-ZJ-AAXF-01A-31R-A42T-07 | TCGA-C5-A1M7-01A-11R-A13Y-07 | TCGA-JW-A5VI-01A-11R-A28H-07 |
| --- | --- | --- | --- | --- | --- | --- | --- | --- | --- |
| ST8SIA6 | 0 | 0 | 145 | 273 | 20 | 21 | 49 | 168 | 16 |
| ST6GALNAC5 | 140 | 5 | 146 | 62 | 260 | 23 | 26 | 189 | 368 |
| ST8SIA3 | 2 | 0 | 0 | 0 | 0 | 0 | 0 | 0 | 0 |
| ST8SIA5 | 22 | 2 | 150 | 18 | 2 | 21 | 2 | 11 | 4 |
| ST6GALNAC1 | 4900 | 984 | 45 | 38 | 1652 | 663 | 110 | 1035 | 83 |
| ST8SIA2 | 107 | 2 | 72 | 1069 | 3 | 40 | 3 | 4 | 17 |
| ST6GAL2 | 26 | 10 | 194 | 6 | 97 | 117 | 104 | 7 | 214 |
| ST6GALNAC3 | 30 | 8 | 60 | 183 | 53 | 100 | 55 | 26 | 27 |
| ST8SIA4 | 218 | 90 | 470 | 711 | 814 | 745 | 258 | 533 | 389 |
| ST3GAL3 | 72 | 217 | 103 | 132 | 116 | 62 | 93 | 152 | 41 |
| ST6GALNAC6 | 24 | 13 | 13 | 9 | 21 | 14 | 10 | 25 | 14 |
| ST6GALNAC2 | 176 | 821 | 485 | 301 | 3610 | 294 | 267 | 715 | 1656 |
| ST3GAL4 | 14651 | 1651 | 646 | 1222 | 2314 | 3306 | 923 | 320 | 1122 |
| ST3GAL6 | 769 | 678 | 329 | 240 | 612 | 357 | 128 | 324 | 260 |
| ST6GAL1 | 7660 | 213 | 2281 | 9790 | 1571 | 2151 | 1600 | 6329 | 1087 |
| ST3GAL2 | 678 | 534 | 1355 | 1412 | 1096 | 1893 | 862 | 926 | 1208 |
| ST3GAL1 | 3788 | 1040 | 2998 | 473 | 2117 | 2283 | 1314 | 6031 | 1910 |
| ST6GALNAC4 | 1249 | 1123 | 1007 | 406 | 2647 | 1444 | 893 | 1736 | 2054 |
| ST8SIA1 | 15 | 97 | 86 | 37 | 107 | 56 | 182 | 89 | 73 |

| ID | TCGA-EA-A411-01A-11R-A24H-07 | TCGA-C5-A1MF-01A-11R-A13Y-07 | TCGA-MA-AA3X-01A-22R-A42S-07 | TCGA-PN-A8MA-01A-11R-A36F-07 | TCGA-EK-A2RB-01A-11R-A18M-07 | TCGA-C5-A7CO-01A-11R-A352-07 | TCGA-HM-A4S6-01A-11R-A26T-07 | TCGA-IR-A3LA-01A-11R-A22U-07 | TCGA-GH-A9DA-01A-21R-A37O-07 |
| --- | --- | --- | --- | --- | --- | --- | --- | --- | --- |
| ST8SIA6 | 154 | 3 | 8 | 4 | 1 | 3 | 3 | 245 | 18 |
| ST6GALNAC5 | 1159 | 151 | 60 | 160 | 23 | 59 | 171 | 239 | 51 |
| ST8SIA3 | 0 | 0 | 1 | 0 | 1 | 0 | 4 | 1 | 0 |
| ST8SIA5 | 12 | 3 | 8 | 15 | 17 | 8 | 0 | 9 | 1 |
| ST6GALNAC1 | 12268 | 201 | 432 | 550 | 184 | 948 | 10 | 2179 | 94 |
| ST8SIA2 | 22 | 16 | 0 | 5 | 2 | 0 | 13 | 3 | 7 |
| ST6GAL2 | 966 | 40 | 19 | 107 | 18 | 30 | 53 | 80 | 21 |
| ST6GALNAC3 | 141 | 14 | 45 | 495 | 13 | 11 | 29 | 23 | 12 |
| ST8SIA4 | 703 | 725 | 507 | 507 | 94 | 182 | 513 | 231 | 248 |
| ST3GAL3 | 196 | 27 | 53 | 146 | 48 | 64 | 104 | 240 | 35 |
| ST6GALNAC6 | 11 | 14 | 5 | 8 | 2 | 73 | 6 | 5 | 6 |
| ST6GALNAC2 | 1595 | 12 | 349 | 482 | 204 | 399 | 366 | 545 | 919 |
| ST3GAL4 | 1365 | 2444 | 828 | 738 | 1020 | 609 | 1096 | 1472 | 2425 |
| ST3GAL6 | 230 | 330 | 267 | 136 | 1108 | 404 | 122 | 1651 | 213 |
| ST6GAL1 | 5518 | 2644 | 679 | 2544 | 91 | 1673 | 691 | 1789 | 1704 |
| ST3GAL2 | 1798 | 356 | 895 | 1251 | 426 | 394 | 448 | 870 | 342 |
| ST3GAL1 | 1829 | 1643 | 4798 | 1552 | 2963 | 2398 | 3747 | 389 | 2620 |
| ST6GALNAC4 | 1315 | 2129 | 1017 | 662 | 214 | 3316 | 1203 | 773 | 1312 |
| ST8SIA1 | 128 | 72 | 117 | 92 | 266 | 62 | 116 | 63 | 44 |

| ID | TCGA-VS-A9UI-01A-11R-A42T-07 | TCGA-EK-A2RN-01A-12R-A213-07 | TCGA-VS-A9V2-01A-11R-A42T-07 | TCGA-VS-A8EB-01A-11R-A36F-07 | TCGA-HM-A6W2-06A-22R-A33Z-07 | TCGA-ZJ-AAXD-01A-21R-A42T-07 | TCGA-IR-A3LK-01A-12R-A213-07 | TCGA-JW-A69B-01A-11R-A32P-07 | TCGA-JW-A5VG-01A-11R-A28H-07 |
| --- | --- | --- | --- | --- | --- | --- | --- | --- | --- |
| ST8SIA6 | 5 | 0 | 27 | 9 | 11 | 75 | 0 | 5 | 12 |
| ST6GALNAC5 | 24 | 12 | 51 | 77 | 40 | 123 | 11 | 60 | 212 |
| ST8SIA3 | 0 | 0 | 0 | 1 | 4 | 1 | 0 | 1 | 0 |
| ST8SIA5 | 1 | 1 | 0 | 0 | 67 | 6 | 9 | 3 | 47 |
| ST6GALNAC1 | 75 | 351 | 1928 | 1 | 93 | 1154 | 60 | 5359 | 151 |
| ST8SIA2 | 2 | 2 | 6 | 15 | 0 | 8 | 4 | 13 | 6 |
| ST6GAL2 | 1 | 7 | 53 | 22 | 33 | 67 | 9 | 162 | 2 |
| ST6GALNAC3 | 5 | 7 | 23 | 6 | 26 | 10 | 14 | 39 | 4 |
| ST8SIA4 | 170 | 308 | 357 | 245 | 94 | 496 | 386 | 360 | 323 |
| ST3GAL3 | 34 | 47 | 60 | 82 | 99 | 31 | 122 | 63 | 87 |
| ST6GALNAC6 | 3 | 8 | 9 | 5 | 49 | 5 | 5 | 24 | 13 |
| ST6GALNAC2 | 424 | 173 | 544 | 514 | 17 | 306 | 374 | 48 | 538 |
| ST3GAL4 | 57 | 684 | 226 | 800 | 995 | 550 | 1256 | 311 | 952 |
| ST3GAL6 | 133 | 1897 | 71 | 60 | 123 | 75 | 104 | 237 | 370 |
| ST6GAL1 | 372 | 511 | 2753 | 968 | 135 | 4678 | 500 | 11200 | 1153 |
| ST3GAL2 | 312 | 221 | 648 | 1391 | 1029 | 443 | 471 | 2625 | 1081 |
| ST3GAL1 | 1865 | 3625 | 1890 | 2003 | 385 | 925 | 528 | 2269 | 3145 |
| ST6GALNAC4 | 532 | 258 | 866 | 656 | 1997 | 648 | 1429 | 4593 | 1257 |
| ST8SIA1 | 48 | 183 | 133 | 499 | 28 | 29 | 27 | 49 | 22 |

| ID | TCGA-JX-A5QV-01A-22R-A28H-07 | TCGA-EK-A2RE-01A-11R-A18M-07 | TCGA-ZJ-AAXN-01A-11R-A42T-07 | TCGA-C5-A7XC-01A-11R-A38B-07 | TCGA-C5-A3HE-01A-21R-A22U-07 | TCGA-DS-A0VK-01A-21R-A10U-07 | TCGA-JX-A3Q8-01A-11R-A21T-07 | TCGA-C5-A3HD-01B-11R-A213-07 | TCGA-EK-A2RK-01A-11R-A18M-07 |
| --- | --- | --- | --- | --- | --- | --- | --- | --- | --- |
| ST8SIA6 | 24 | 79 | 44 | 15 | 29 | 101 | 13 | 135 | 4 |
| ST6GALNAC5 | 13 | 111 | 253 | 111 | 67 | 144 | 5 | 3 | 46 |
| ST8SIA3 | 1 | 0 | 0 | 0 | 6 | 1 | 0 | 2 | 1 |
| ST8SIA5 | 2 | 5 | 4 | 15 | 27 | 2 | 17 | 9 | 4 |
| ST6GALNAC1 | 146 | 654 | 328 | 3552 | 2583 | 1934 | 4631 | 808 | 675 |
| ST8SIA2 | 938 | 17 | 24 | 11 | 22 | 6 | 3 | 7 | 3 |
| ST6GAL2 | 7 | 54 | 55 | 122 | 48 | 191 | 14 | 2 | 35 |
| ST6GALNAC3 | 10 | 23 | 37 | 19 | 42 | 70 | 9 | 5 | 25 |
| ST8SIA4 | 201 | 449 | 858 | 465 | 701 | 293 | 292 | 73 | 228 |
| ST3GAL3 | 49 | 52 | 117 | 61 | 56 | 89 | 97 | 90 | 123 |
| ST6GALNAC6 | 0 | 11 | 10 | 21 | 156 | 4 | 10 | 6 | 6 |
| ST6GALNAC2 | 335 | 592 | 712 | 558 | 46 | 711 | 792 | 338 | 168 |
| ST3GAL4 | 1488 | 2279 | 1166 | 904 | 5638 | 3395 | 582 | 944 | 229 |
| ST3GAL6 | 549 | 882 | 305 | 374 | 228 | 83 | 183 | 395 | 189 |
| ST6GAL1 | 3173 | 247 | 3912 | 6782 | 14283 | 6059 | 6931 | 1271 | 757 |
| ST3GAL2 | 303 | 655 | 943 | 831 | 776 | 916 | 1216 | 378 | 716 |
| ST3GAL1 | 2753 | 3052 | 5189 | 3461 | 2438 | 9003 | 584 | 3909 | 2296 |
| ST6GALNAC4 | 520 | 1784 | 1327 | 1375 | 6465 | 2471 | 2349 | 1172 | 1742 |
| ST8SIA1 | 75 | 55 | 112 | 116 | 96 | 44 | 51 | 13 | 37 |

| ID | TCGA-VS-A8EI-01A-11R-A37O-07 | TCGA-ZJ-A8QO-01A-11R-A37O-07 | TCGA-DG-A2KK-01A-11R-A180-07 | TCGA-R2-A69V-01A-11R-A32P-07 | TCGA-C5-A7CJ-01A-11R-A32P-07 | TCGA-FU-A3WB-01A-11R-A22U-07 | TCGA-EK-A3GM-01A-11R-A213-07 | TCGA-VS-A9UT-01A-11R-A42T-07 | TCGA-EA-A50E-01A-21R-A26T-07 |
| --- | --- | --- | --- | --- | --- | --- | --- | --- | --- |
| ST8SIA6 | 20 | 19 | 0 | 10 | 18 | 86 | 1 | 79 | 7 |
| ST6GALNAC5 | 34 | 42 | 23 | 74 | 196 | 88 | 21 | 214 | 200 |
| ST8SIA3 | 1 | 1 | 0 | 0 | 0 | 1 | 0 | 160 | 0 |
| ST8SIA5 | 3 | 3 | 36 | 19 | 7 | 1 | 1 | 134 | 33 |
| ST6GALNAC1 | 503 | 67 | 2361 | 465 | 769 | 625 | 7114 | 22 | 865 |
| ST8SIA2 | 3 | 121 | 16 | 3 | 33 | 329 | 7 | 489 | 0 |
| ST6GAL2 | 124 | 88 | 33 | 30 | 215 | 316 | 6 | 151 | 155 |
| ST6GALNAC3 | 30 | 46 | 16 | 34 | 67 | 29 | 12 | 457 | 77 |
| ST8SIA4 | 280 | 717 | 282 | 787 | 642 | 242 | 107 | 1000 | 509 |
| ST3GAL3 | 115 | 111 | 133 | 20 | 60 | 142 | 44 | 73 | 40 |
| ST6GALNAC6 | 20 | 20 | 37 | 8 | 12 | 12 | 20 | 22 | 5 |
| ST6GALNAC2 | 431 | 714 | 536 | 100 | 864 | 1803 | 143 | 39 | 472 |
| ST3GAL4 | 791 | 1480 | 1667 | 534 | 1099 | 1288 | 123 | 381 | 494 |
| ST3GAL6 | 398 | 451 | 317 | 176 | 404 | 489 | 32 | 148 | 507 |
| ST6GAL1 | 3842 | 3329 | 20225 | 1944 | 2269 | 2574 | 40296 | 9164 | 714 |
| ST3GAL2 | 887 | 2190 | 1320 | 602 | 878 | 1555 | 1278 | 2825 | 752 |
| ST3GAL1 | 868 | 1957 | 2287 | 2036 | 4760 | 750 | 219 | 2882 | 2215 |
| ST6GALNAC4 | 1690 | 1747 | 4088 | 855 | 618 | 1567 | 1627 | 1588 | 767 |
| ST8SIA1 | 100 | 305 | 85 | 103 | 145 | 230 | 27 | 298 | 71 |

| ID | TCGA-C5-A1BE-01B-11R-A13Y-07 | TCGA-EK-A2PI-01A-11R-A18M-07 | TCGA-UC-A7PG-01A-11R-A42S-07 | TCGA-ZJ-AAXA-01A-11R-A42T-07 | TCGA-4J-AA1J-01A-21R-A38B-07 | TCGA-VS-A8EJ-01A-11R-A37O-07 | TCGA-VS-A9UB-01A-22R-A42T-07 | TCGA-C5-A3HL-01A-11R-A213-07 | TCGA-MA-AA42-01A-12R-A38B-07 |
| --- | --- | --- | --- | --- | --- | --- | --- | --- | --- |
| ST8SIA6 | 94 | 61 | 55 | 5 | 253 | 3 | 15 | 329 | 10 |
| ST6GALNAC5 | 91 | 84 | 59 | 102 | 133 | 162 | 405 | 27 | 47 |
| ST8SIA3 | 0 | 0 | 0 | 1 | 0 | 0 | 0 | 0 | 0 |
| ST8SIA5 | 6 | 13 | 3 | 8 | 6 | 28 | 0 | 6 | 1 |
| ST6GALNAC1 | 3721 | 1276 | 459 | 1372 | 1391 | 925 | 374 | 1205 | 50 |
| ST8SIA2 | 10 | 90 | 5 | 6 | 10 | 250 | 2 | 162 | 0 |
| ST6GAL2 | 184 | 376 | 75 | 243 | 155 | 9 | 0 | 79 | 9 |
| ST6GALNAC3 | 25 | 55 | 14 | 39 | 73 | 95 | 17 | 30 | 47 |
| ST8SIA4 | 289 | 207 | 253 | 273 | 194 | 447 | 662 | 1098 | 1150 |
| ST3GAL3 | 79 | 167 | 82 | 137 | 58 | 89 | 69 | 45 | 18 |
| ST6GALNAC6 | 11 | 13 | 7 | 19 | 8 | 4 | 11 | 7 | 9 |
| ST6GALNAC2 | 1248 | 1380 | 945 | 2326 | 325 | 112 | 673 | 1282 | 1098 |
| ST3GAL4 | 415 | 1180 | 197 | 546 | 2336 | 640 | 935 | 418 | 1123 |
| ST3GAL6 | 332 | 544 | 283 | 188 | 55 | 478 | 102 | 403 | 461 |
| ST6GAL1 | 830 | 403 | 363 | 403 | 3183 | 4357 | 3578 | 8729 | 1696 |
| ST3GAL2 | 743 | 796 | 478 | 1025 | 656 | 948 | 837 | 784 | 1300 |
| ST3GAL1 | 4373 | 3247 | 4327 | 8371 | 874 | 1998 | 4395 | 7345 | 4964 |
| ST6GALNAC4 | 1033 | 1428 | 686 | 3737 | 1149 | 779 | 747 | 1716 | 1579 |
| ST8SIA1 | 82 | 53 | 249 | 181 | 93 | 43 | 49 | 63 | 449 |

| ID | TCGA-FU-A5XV-01A-11R-A28H-07 | TCGA-MU-A8JM-01A-11R-A36F-07 | TCGA-EA-A44S-01A-12R-A26T-07 | TCGA-LP-A4AU-01A-32R-A32Y-07 | TCGA-C5-A901-01A-11R-A37O-07 | TCGA-ZX-AA5X-01A-11R-A42T-07 | TCGA-VS-A94Y-01A-11R-A38B-07 | TCGA-JX-A3Q0-01A-11R-A32Y-07 | TCGA-DS-A0VN-01A-21R-A10U-07 |
| --- | --- | --- | --- | --- | --- | --- | --- | --- | --- |
| ST8SIA6 | 47 | 380 | 11 | 185 | 60 | 43 | 11 | 48 | 33 |
| ST6GALNAC5 | 74 | 112 | 52 | 56 | 136 | 76 | 61 | 25 | 298 |
| ST8SIA3 | 4 | 0 | 0 | 1 | 3 | 0 | 0 | 0 | 2 |
| ST8SIA5 | 4 | 1 | 5 | 2 | 6 | 1 | 3 | 1 | 15 |
| ST6GALNAC1 | 2584 | 46 | 160 | 1992 | 154 | 504 | 275 | 115 | 313 |
| ST8SIA2 | 0 | 9 | 4 | 13 | 4 | 10 | 22 | 1 | 30 |
| ST6GAL2 | 20 | 29 | 142 | 34 | 107 | 49 | 20 | 12 | 36 |
| ST6GALNAC3 | 21 | 16 | 18 | 47 | 37 | 48 | 14 | 13 | 80 |
| ST8SIA4 | 196 | 225 | 345 | 393 | 251 | 307 | 132 | 413 | 934 |
| ST3GAL3 | 90 | 77 | 42 | 661 | 85 | 45 | 77 | 93 | 73 |
| ST6GALNAC6 | 7 | 6 | 8 | 58 | 8 | 6 | 4 | 8 | 17 |
| ST6GALNAC2 | 797 | 904 | 429 | 479 | 771 | 305 | 265 | 736 | 476 |
| ST3GAL4 | 1306 | 995 | 1373 | 2640 | 264 | 739 | 4725 | 578 | 1171 |
| ST3GAL6 | 223 | 1374 | 397 | 241 | 619 | 156 | 185 | 141 | 236 |
| ST6GAL1 | 9522 | 4690 | 483 | 1973 | 862 | 1797 | 871 | 877 | 1146 |
| ST3GAL2 | 269 | 521 | 679 | 627 | 480 | 710 | 897 | 516 | 1268 |
| ST3GAL1 | 709 | 3942 | 2318 | 4221 | 4931 | 1839 | 4407 | 2452 | 1730 |
| ST6GALNAC4 | 1162 | 1012 | 334 | 4516 | 1197 | 965 | 788 | 1337 | 1305 |
| ST8SIA1 | 62 | 45 | 175 | 44 | 51 | 85 | 35 | 70 | 147 |

| ID | TCGA-C5-A8XK-01A-11R-A37O-07 | TCGA-MU-A51Y-01A-11R-A26T-07 | TCGA-VS-A9UO-01A-11R-A42T-07 | TCGA-BI-A0VR-01A-11R-A10U-07 | TCGA-C5-A8YR-01A-12R-A37O-07 | TCGA-VS-A8EC-01A-11R-A36F-07 | TCGA-EK-A2PL-01A-11R-A18M-07 | TCGA-MA-AA43-01A-11R-A42T-07 | TCGA-VS-A959-01A-11R-A42T-07 |
| --- | --- | --- | --- | --- | --- | --- | --- | --- | --- |
| ST8SIA6 | 0 | 15 | 3 | 24 | 1 | 12 | 32 | 85 | 13 |
| ST6GALNAC5 | 16 | 47 | 7 | 179 | 18 | 3 | 52 | 10 | 77 |
| ST8SIA3 | 1 | 1 | 1 | 0 | 2 | 0 | 0 | 0 | 0 |
| ST8SIA5 | 4 | 6 | 44 | 1 | 1 | 1 | 0 | 75 | 5 |
| ST6GALNAC1 | 208 | 2803 | 10760 | 1074 | 92 | 718 | 422 | 55 | 1590 |
| ST8SIA2 | 2 | 2 | 8 | 14 | 0 | 0 | 0 | 107 | 44 |
| ST6GAL2 | 13 | 160 | 9 | 62 | 2 | 186 | 16 | 98 | 25 |
| ST6GALNAC3 | 25 | 61 | 13 | 47 | 22 | 19 | 2 | 99 | 16 |
| ST8SIA4 | 249 | 577 | 106 | 611 | 516 | 90 | 100 | 400 | 306 |
| ST3GAL3 | 117 | 59 | 141 | 82 | 180 | 398 | 26 | 70 | 86 |
| ST6GALNAC6 | 8 | 15 | 605 | 15 | 16 | 6 | 6 | 11 | 16 |
| ST6GALNAC2 | 372 | 391 | 638 | 711 | 138 | 734 | 452 | 305 | 117 |
| ST3GAL4 | 1338 | 472 | 7536 | 1691 | 4313 | 846 | 3911 | 2205 | 1663 |
| ST3GAL6 | 124 | 303 | 304 | 351 | 166 | 977 | 428 | 396 | 356 |
| ST6GAL1 | 1992 | 2370 | 34911 | 9507 | 213 | 111 | 1857 | 4103 | 28998 |
| ST3GAL2 | 844 | 782 | 927 | 894 | 1487 | 528 | 208 | 2694 | 718 |
| ST3GAL1 | 2988 | 3868 | 4306 | 2564 | 4451 | 2934 | 697 | 1894 | 1894 |
| ST6GALNAC4 | 1070 | 931 | 31012 | 2046 | 958 | 679 | 705 | 764 | 1053 |
| ST8SIA1 | 18 | 84 | 43 | 216 | 26 | 8 | 27 | 16 | 72 |

| ID | TCGA-EK-A2PG-01A-11R-A18M-07 | TCGA-C5-A1MH-01A-11R-A14Y-07 | TCGA-C5-A1M8-01A-21R-A13Y-07 | TCGA-EK-A3GJ-01A-21R-A213-07 | TCGA-DG-A2KJ-01A-11R-A32Y-07 | TCGA-Q1-A5R1-01A-11R-A28H-07 | TCGA-VS-A954-01A-11R-A38B-07 | TCGA-EA-A43B-01A-81R-A32Y-07 | TCGA-VS-A8EL-01A-11R-A37O-07 |
| --- | --- | --- | --- | --- | --- | --- | --- | --- | --- |
| ST8SIA6 | 15 | 266 | 106 | 1 | 2 | 14 | 10 | 11 | 4 |
| ST6GALNAC5 | 3 | 74 | 35 | 13 | 75 | 11 | 48 | 40 | 56 |
| ST8SIA3 | 3 | 0 | 1 | 0 | 3 | 1 | 0 | 0 | 1 |
| ST8SIA5 | 13 | 3 | 30 | 1 | 1 | 18 | 7 | 2 | 0 |
| ST6GALNAC1 | 560 | 1342 | 164 | 25 | 414 | 4832 | 60 | 19 | 151 |
| ST8SIA2 | 0 | 2 | 7 | 6 | 16 | 2 | 5 | 6 | 5 |
| ST6GAL2 | 0 | 81 | 77 | 3 | 20 | 15 | 62 | 45 | 28 |
| ST6GALNAC3 | 7 | 81 | 29 | 10 | 7 | 29 | 163 | 21 | 34 |
| ST8SIA4 | 128 | 997 | 139 | 573 | 104 | 149 | 173 | 222 | 628 |
| ST3GAL3 | 91 | 67 | 48 | 53 | 16 | 57 | 42 | 19 | 84 |
| ST6GALNAC6 | 4 | 10 | 9 | 10 | 5 | 27 | 19 | 4 | 5 |
| ST6GALNAC2 | 384 | 794 | 1464 | 1184 | 11 | 456 | 591 | 86 | 891 |
| ST3GAL4 | 126 | 810 | 435 | 319 | 651 | 1649 | 209 | 663 | 874 |
| ST3GAL6 | 76 | 363 | 358 | 75 | 134 | 583 | 571 | 74 | 1799 |
| ST6GAL1 | 172 | 2003 | 222 | 532 | 463 | 18604 | 358 | 816 | 1270 |
| ST3GAL2 | 1024 | 1055 | 770 | 503 | 423 | 940 | 492 | 451 | 829 |
| ST3GAL1 | 4019 | 4865 | 4494 | 1676 | 1000 | 1331 | 2058 | 887 | 3187 |
| ST6GALNAC4 | 359 | 1565 | 508 | 1129 | 757 | 2452 | 1605 | 552 | 1015 |
| ST8SIA1 | 426 | 216 | 239 | 55 | 17 | 37 | 153 | 21 | 367 |

| ID | TCGA-VS-A9V4-01A-12R-A42T-07 | TCGA-JW-A852-01A-11R-A352-07 | TCGA-C5-A7UI-01A-11R-A36F-07 | TCGA-VS-A9UJ-01A-11R-A42T-07 | TCGA-EA-A4BA-01A-21R-A26T-07 | TCGA-UC-A7PI-01A-11R-A42S-07 | TCGA-Q1-A6DV-01A-11R-A32P-07 | TCGA-ZJ-AAX4-01A-11R-A42T-07 | TCGA-C5-A1ME-01A-11R-A13Y-07 |
| --- | --- | --- | --- | --- | --- | --- | --- | --- | --- |
| ST8SIA6 | 33 | 54 | 2 | 2 | 5 | 167 | 208 | 17 | 6 |
| ST6GALNAC5 | 23 | 10 | 29 | 37 | 168 | 8 | 42 | 27 | 18 |
| ST8SIA3 | 0 | 0 | 0 | 0 | 1 | 2 | 2 | 0 | 0 |
| ST8SIA5 | 0 | 2 | 1 | 9 | 5 | 8 | 10 | 31 | 8 |
| ST6GALNAC1 | 623 | 465 | 29 | 925 | 1575 | 7112 | 4202 | 96 | 3179 |
| ST8SIA2 | 4 | 0 | 3 | 1403 | 127 | 1 | 131 | 19 | 98 |
| ST6GAL2 | 11 | 11 | 9 | 353 | 498 | 45 | 7 | 35 | 13 |
| ST6GALNAC3 | 6 | 4 | 10 | 11 | 31 | 43 | 12 | 19 | 18 |
| ST8SIA4 | 46 | 78 | 170 | 39 | 5873 | 96 | 1841 | 351 | 205 |
| ST3GAL3 | 56 | 36 | 52 | 37 | 42 | 115 | 72 | 58 | 43 |
| ST6GALNAC6 | 7 | 8 | 7 | 11 | 8 | 43 | 9 | 6 | 5 |
| ST6GALNAC2 | 10 | 75 | 83 | 824 | 29 | 555 | 1269 | 602 | 42 |
| ST3GAL4 | 898 | 513 | 722 | 718 | 571 | 3802 | 778 | 484 | 3021 |
| ST3GAL6 | 5 | 121 | 58 | 104 | 597 | 177 | 360 | 285 | 135 |
| ST6GAL1 | 849 | 3224 | 930 | 3591 | 1972 | 21493 | 7626 | 698 | 1700 |
| ST3GAL2 | 666 | 333 | 365 | 1424 | 1539 | 1058 | 789 | 493 | 851 |
| ST3GAL1 | 236 | 1166 | 423 | 2488 | 5282 | 2077 | 336 | 3446 | 1337 |
| ST6GALNAC4 | 301 | 1389 | 798 | 988 | 870 | 4052 | 1068 | 444 | 2265 |
| ST8SIA1 | 203 | 8 | 690 | 8 | 438 | 27 | 37 | 91 | 73 |

| ID | TCGA-DS-A0VL-01A-21R-A10U-07 | TCGA-MY-A5BF-01A-11R-A26T-07 | TCGA-VS-A94Z-01A-11R-A38B-07 | TCGA-JW-A5VL-01A-11R-A28H-07 | TCGA-IR-A3LB-01A-11R-A24H-07 | TCGA-MA-AA3W-01A-11R-A38B-07 | TCGA-MY-A5BE-01A-21R-A26T-07 | TCGA-DS-A5RQ-01A-11R-A28H-07 | TCGA-LP-A4AV-01A-11R-A32Y-07 |
| --- | --- | --- | --- | --- | --- | --- | --- | --- | --- |
| ST8SIA6 | 103 | 6 | 16 | 0 | 21 | 154 | 31 | 2 | 2 |
| ST6GALNAC5 | 43 | 9 | 24 | 28 | 447 | 22 | 77 | 2 | 44 |
| ST8SIA3 | 0 | 0 | 0 | 0 | 0 | 0 | 0 | 1 | 0 |
| ST8SIA5 | 6 | 10 | 48 | 8 | 79 | 1 | 5 | 2 | 7 |
| ST6GALNAC1 | 2209 | 90 | 238 | 15 | 3410 | 1266 | 202 | 267 | 224 |
| ST8SIA2 | 5 | 2 | 3 | 4 | 302 | 0 | 1 | 0 | 5 |
| ST6GAL2 | 14 | 7 | 92 | 12 | 557 | 7 | 72 | 77 | 20 |
| ST6GALNAC3 | 27 | 38 | 53 | 11 | 49 | 26 | 67 | 18 | 15 |
| ST8SIA4 | 365 | 422 | 1154 | 473 | 369 | 685 | 1640 | 231 | 162 |
| ST3GAL3 | 76 | 21 | 66 | 50 | 131 | 58 | 53 | 23 | 70 |
| ST6GALNAC6 | 13 | 6 | 18 | 24 | 32 | 15 | 43 | 3 | 7 |
| ST6GALNAC2 | 1601 | 391 | 522 | 398 | 110 | 479 | 247 | 495 | 16 |
| ST3GAL4 | 511 | 1649 | 361 | 1256 | 4163 | 870 | 527 | 460 | 621 |
| ST3GAL6 | 72 | 133 | 411 | 141 | 814 | 106 | 127 | 122 | 99 |
| ST6GAL1 | 17222 | 305 | 2150 | 507 | 2092 | 7630 | 3639 | 3457 | 364 |
| ST3GAL2 | 613 | 329 | 1487 | 579 | 1097 | 581 | 1269 | 537 | 429 |
| ST3GAL1 | 5006 | 3072 | 4662 | 4803 | 4886 | 1354 | 1901 | 2058 | 270 |
| ST6GALNAC4 | 2380 | 563 | 1807 | 2234 | 1674 | 1542 | 1459 | 1043 | 1484 |
| ST8SIA1 | 37 | 47 | 163 | 207 | 190 | 91 | 402 | 63 | 108 |

| ID | TCGA-VS-A9U6-01A-11R-A42T-07 | TCGA-MY-A5BD-01A-11R-A26T-07 | TCGA-EK-A2IR-01A-11R-A180-07 | TCGA-VS-A9UM-01A-11R-A42T-07 | TCGA-C5-A3HF-01A-11R-A213-07 | TCGA-RA-A741-01A-11R-A33Z-07 | TCGA-DG-A2KL-01A-11R-A180-07 | TCGA-C5-A7X5-01A-11R-A36F-07 | TCGA-DG-A2KH-01A-21R-A22U-07 |
| --- | --- | --- | --- | --- | --- | --- | --- | --- | --- |
| ST8SIA6 | 50 | 2 | 1 | 29 | 72 | 22 | 372 | 13 | 242 |
| ST6GALNAC5 | 120 | 0 | 57 | 123 | 119 | 30 | 1160 | 71 | 9 |
| ST8SIA3 | 0 | 0 | 1 | 0 | 7 | 2 | 0 | 0 | 17 |
| ST8SIA5 | 13 | 11 | 5 | 10 | 2 | 6 | 5 | 11 | 6 |
| ST6GALNAC1 | 153 | 214 | 570 | 203 | 9685 | 2115 | 1214 | 41 | 7955 |
| ST8SIA2 | 149 | 93 | 6 | 11 | 40 | 0 | 219 | 5 | 704 |
| ST6GAL2 | 54 | 2 | 6 | 119 | 327 | 37 | 394 | 10 | 3 |
| ST6GALNAC3 | 23 | 20 | 20 | 26 | 127 | 15 | 58 | 24 | 11 |
| ST8SIA4 | 416 | 96 | 110 | 364 | 467 | 309 | 519 | 102 | 151 |
| ST3GAL3 | 218 | 49 | 262 | 68 | 53 | 48 | 89 | 72 | 97 |
| ST6GALNAC6 | 13 | 11 | 6 | 5 | 63 | 4 | 13 | 3 | 82 |
| ST6GALNAC2 | 797 | 429 | 1756 | 1102 | 312 | 410 | 1000 | 994 | 184 |
| ST3GAL4 | 817 | 1138 | 1515 | 439 | 2688 | 1224 | 1526 | 1553 | 5764 |
| ST3GAL6 | 483 | 449 | 1328 | 296 | 285 | 106 | 302 | 412 | 357 |
| ST6GAL1 | 9413 | 9312 | 150 | 1417 | 16204 | 5422 | 3649 | 4045 | 55314 |
| ST3GAL2 | 1109 | 732 | 605 | 615 | 973 | 953 | 1135 | 746 | 588 |
| ST3GAL1 | 2437 | 836 | 21710 | 2559 | 4304 | 4760 | 3744 | 2672 | 2917 |
| ST6GALNAC4 | 817 | 1400 | 712 | 861 | 4721 | 1915 | 918 | 973 | 4763 |
| ST8SIA1 | 73 | 126 | 56 | 70 | 125 | 63 | 188 | 177 | 20 |

| ID | TCGA-C5-A7CK-01A-11R-A32P-07 | TCGA-C5-A8ZZ-01A-11R-A37O-07 | TCGA-C5-A7CL-01A-11R-A32P-07 | TCGA-JW-A5VH-01A-11R-A28H-07 | TCGA-EA-A5ZF-01A-11R-A28H-07 | TCGA-Q1-A5R2-01A-11R-A28H-07 | TCGA-VS-A9U7-01A-11R-A42T-07 | TCGA-VS-A9V1-01A-11R-A42T-07 | TCGA-C5-A0TN-01A-21R-A14Y-07 |
| --- | --- | --- | --- | --- | --- | --- | --- | --- | --- |
| ST8SIA6 | 4 | 315 | 23 | 303 | 11 | 44 | 363 | 24 | 2 |
| ST6GALNAC5 | 295 | 727 | 74 | 140 | 211 | 48 | 20 | 23 | 15 |
| ST8SIA3 | 0 | 1 | 1 | 0 | 0 | 1 | 0 | 1 | 0 |
| ST8SIA5 | 8 | 1 | 2 | 5 | 13 | 8 | 10 | 3 | 27 |
| ST6GALNAC1 | 2141 | 1231 | 663 | 52 | 7505 | 912 | 75 | 2017 | 27 |
| ST8SIA2 | 70 | 78 | 17 | 56 | 59 | 4 | 14 | 1 | 2 |
| ST6GAL2 | 468 | 3007 | 49 | 4199 | 228 | 35 | 77 | 3 | 8 |
| ST6GALNAC3 | 33 | 166 | 26 | 260 | 219 | 15 | 17 | 10 | 12 |
| ST8SIA4 | 174 | 241 | 227 | 229 | 318 | 270 | 493 | 75 | 155 |
| ST3GAL3 | 96 | 312 | 99 | 77 | 289 | 38 | 72 | 108 | 51 |
| ST6GALNAC6 | 22 | 11 | 8 | 4 | 15 | 10 | 14 | 26 | 1 |
| ST6GALNAC2 | 1268 | 1184 | 540 | 326 | 872 | 143 | 851 | 58 | 405 |
| ST3GAL4 | 1168 | 1490 | 1559 | 1413 | 1038 | 572 | 458 | 5653 | 493 |
| ST3GAL6 | 462 | 188 | 187 | 195 | 458 | 307 | 191 | 86 | 167 |
| ST6GAL1 | 2353 | 554 | 1600 | 1079 | 29853 | 1271 | 2541 | 4917 | 578 |
| ST3GAL2 | 824 | 937 | 756 | 938 | 1105 | 799 | 1151 | 497 | 1045 |
| ST3GAL1 | 1716 | 4898 | 2086 | 452 | 812 | 2224 | 4023 | 3864 | 372 |
| ST6GALNAC4 | 1107 | 807 | 930 | 454 | 1130 | 1464 | 1881 | 3440 | 970 |
| ST8SIA1 | 64 | 111 | 60 | 29 | 37 | 134 | 97 | 11 | 14 |

| ID | TCGA-EA-A3QD-01A-32R-A22U-07 | TCGA-DS-A0VM-01A-11R-A10U-07 | TCGA-EA-A439-01A-11R-A24H-07 | TCGA-EA-A1QT-01A-11R-A14Y-07 | TCGA-C5-A8XI-01A-11R-A37O-07 | TCGA-VS-A8QF-01A-21R-A37O-07 | TCGA-EA-A5ZD-01A-11R-A28H-07 | TCGA-DR-A0ZL-01A-11R-A10U-07 | TCGA-C5-A1ML-01A-11R-A14Y-07 |
| --- | --- | --- | --- | --- | --- | --- | --- | --- | --- |
| ST8SIA6 | 22 | 9 | 259 | 56 | 347 | 1 | 56 | 2 | 102 |
| ST6GALNAC5 | 115 | 334 | 1043 | 81 | 24 | 53 | 65 | 21 | 15 |
| ST8SIA3 | 0 | 1 | 1 | 0 | 0 | 2 | 0 | 1 | 0 |
| ST8SIA5 | 6 | 3 | 115 | 2 | 8 | 1 | 5 | 1 | 20 |
| ST6GALNAC1 | 174 | 1789 | 10924 | 4139 | 2008 | 671 | 2093 | 2082 | 507 |
| ST8SIA2 | 12 | 38 | 142 | 2 | 7 | 1 | 2 | 4 | 67 |
| ST6GAL2 | 107 | 172 | 536 | 91 | 21 | 112 | 60 | 8 | 82 |
| ST6GALNAC3 | 103 | 15 | 38 | 28 | 24 | 5 | 44 | 22 | 33 |
| ST8SIA4 | 1175 | 233 | 7604 | 308 | 362 | 322 | 403 | 125 | 764 |
| ST3GAL3 | 56 | 62 | 421 | 175 | 72 | 107 | 61 | 90 | 79 |
| ST6GALNAC6 | 23 | 12 | 9 | 9 | 13 | 10 | 8 | 22 | 9 |
| ST6GALNAC2 | 412 | 930 | 759 | 2076 | 1458 | 529 | 950 | 933 | 1257 |
| ST3GAL4 | 727 | 6429 | 2604 | 427 | 1670 | 830 | 1307 | 451 | 534 |
| ST3GAL6 | 579 | 277 | 251 | 324 | 237 | 137 | 338 | 33 | 503 |
| ST6GAL1 | 2568 | 1161 | 23914 | 258 | 14940 | 1015 | 1058 | 11007 | 6619 |
| ST3GAL2 | 1284 | 1401 | 1714 | 1059 | 951 | 641 | 507 | 367 | 544 |
| ST3GAL1 | 2401 | 5850 | 748 | 4057 | 1050 | 2403 | 2985 | 579 | 2575 |
| ST6GALNAC4 | 2298 | 2434 | 1110 | 1783 | 999 | 1015 | 1830 | 2858 | 761 |
| ST8SIA1 | 416 | 45 | 189 | 63 | 356 | 82 | 59 | 73 | 41 |

| ID | TCGA-EA-A3HU-01A-11R-A213-07 | TCGA-MU-A5YI-01A-11R-A32P-07 | TCGA-C5-A8YT-01A-11R-A37O-07 | TCGA-UC-A7PD-01A-11R-A352-07 | TCGA-Q1-A6DW-01A-11R-A32P-07 | TCGA-C5-A2LY-01A-31R-A18M-07 | TCGA-XS-A8TJ-01A-11R-A36F-07 | TCGA-VS-A9V0-01A-11R-A42T-07 | TCGA-FU-A57G-01A-11R-A26T-07 |
| --- | --- | --- | --- | --- | --- | --- | --- | --- | --- |
| ST8SIA6 | 9 | 21 | 50 | 11 | 15 | 2 | 51 | 1 | 34 |
| ST6GALNAC5 | 119 | 36 | 48 | 3 | 52 | 21 | 138 | 8 | 2021 |
| ST8SIA3 | 1 | 0 | 1 | 0 | 0 | 0 | 0 | 1 | 0 |
| ST8SIA5 | 16 | 2 | 19 | 3 | 9 | 8 | 1 | 10 | 37 |
| ST6GALNAC1 | 110 | 1776 | 3666 | 557 | 63 | 129 | 5536 | 234 | 102 |
| ST8SIA2 | 18 | 2 | 283 | 4 | 8 | 1 | 2 | 4 | 282 |
| ST6GAL2 | 39 | 350 | 3326 | 5 | 20 | 27 | 117 | 6 | 889 |
| ST6GALNAC3 | 81 | 132 | 1347 | 11 | 78 | 15 | 40 | 38 | 222 |
| ST8SIA4 | 2234 | 430 | 268 | 108 | 302 | 438 | 243 | 311 | 5187 |
| ST3GAL3 | 49 | 81 | 93 | 37 | 61 | 24 | 100 | 55 | 30 |
| ST6GALNAC6 | 13 | 1 | 23 | 12 | 27 | 14 | 6 | 2 | 5 |
| ST6GALNAC2 | 498 | 754 | 309 | 936 | 588 | 124 | 970 | 24 | 212 |
| ST3GAL4 | 409 | 1027 | 1544 | 682 | 300 | 436 | 370 | 410 | 1929 |
| ST3GAL6 | 603 | 219 | 589 | 361 | 161 | 155 | 447 | 253 | 247 |
| ST6GAL1 | 3472 | 2477 | 2076 | 1539 | 2257 | 2519 | 6147 | 2284 | 11804 |
| ST3GAL2 | 1032 | 971 | 3112 | 868 | 605 | 467 | 614 | 350 | 1812 |
| ST3GAL1 | 3389 | 1863 | 2178 | 1919 | 2830 | 608 | 4662 | 3222 | 2265 |
| ST6GALNAC4 | 1195 | 897 | 1642 | 1471 | 2196 | 1109 | 506 | 434 | 733 |
| ST8SIA1 | 138 | 183 | 192 | 27 | 35 | 55 | 61 | 74 | 144 |

| ID | TCGA-EA-A97N-01A-11R-A38B-07 | TCGA-EX-A1H5-01A-31R-A13Y-07 | TCGA-MA-AA41-01A-11R-A38B-07 | TCGA-C5-A1MI-01A-11R-A14Y-07 | TCGA-LP-A4AW-01A-11R-A24H-07 | TCGA-C5-A1M5-01A-11R-A13Y-07 | TCGA-C5-A1M6-01A-11R-A13Y-07 | TCGA-VS-A9V3-01A-11R-A42T-07 | TCGA-FU-A770-01A-11R-A33Z-07 |
| --- | --- | --- | --- | --- | --- | --- | --- | --- | --- |
| ST8SIA6 | 97 | 16 | 1 | 480 | 50 | 7 | 65 | 3 | 5 |
| ST6GALNAC5 | 295 | 13 | 15 | 197 | 249 | 170 | 142 | 40 | 30 |
| ST8SIA3 | 2 | 0 | 0 | 0 | 2 | 0 | 0 | 0 | 89 |
| ST8SIA5 | 6 | 20 | 6 | 16 | 3 | 3 | 5 | 0 | 4 |
| ST6GALNAC1 | 1342 | 2999 | 254 | 7200 | 1512 | 4387 | 11955 | 117 | 5432 |
| ST8SIA2 | 10 | 38 | 4 | 282 | 5 | 11 | 61 | 1 | 8 |
| ST6GAL2 | 288 | 38 | 15 | 182 | 48 | 118 | 52 | 17 | 47 |
| ST6GALNAC3 | 28 | 30 | 9 | 76 | 182 | 24 | 367 | 18 | 15 |
| ST8SIA4 | 170 | 257 | 140 | 560 | 235 | 476 | 153 | 262 | 162 |
| ST3GAL3 | 149 | 75 | 47 | 96 | 94 | 76 | 115 | 159 | 65 |
| ST6GALNAC6 | 8 | 20 | 9 | 13 | 10 | 5 | 18 | 13 | 23 |
| ST6GALNAC2 | 796 | 1274 | 947 | 1977 | 514 | 1193 | 273 | 659 | 371 |
| ST3GAL4 | 655 | 1180 | 1054 | 3452 | 931 | 1001 | 1124 | 1269 | 4868 |
| ST3GAL6 | 47 | 470 | 256 | 134 | 193 | 502 | 263 | 144 | 383 |
| ST6GAL1 | 3396 | 4356 | 208 | 12569 | 2251 | 3464 | 33398 | 564 | 24172 |
| ST3GAL2 | 792 | 1222 | 666 | 1443 | 1149 | 937 | 1712 | 523 | 442 |
| ST3GAL1 | 2853 | 585 | 632 | 2059 | 4026 | 3994 | 1428 | 3957 | 2271 |
| ST6GALNAC4 | 508 | 2081 | 363 | 1211 | 1077 | 1163 | 1267 | 1334 | 3346 |
| ST8SIA1 | 61 | 52 | 267 | 204 | 174 | 154 | 55 | 52 | 18 |

| ID | TCGA-EX-A3L1-01A-11R-A32Y-07 | TCGA-C5-A905-01A-11R-A37O-07 | TCGA-EA-A1QS-01A-61R-A22U-07 |
| --- | --- | --- | --- |
| ST8SIA6 | 3 | 2 | 29 |
| ST6GALNAC5 | 93 | 19 | 1315 |
| ST8SIA3 | 1 | 0 | 0 |
| ST8SIA5 | 5 | 5 | 10 |
| ST6GALNAC1 | 1599 | 1428 | 230 |
| ST8SIA2 | 16 | 12 | 72 |
| ST6GAL2 | 21 | 10 | 1358 |
| ST6GALNAC3 | 63 | 12 | 108 |
| ST8SIA4 | 403 | 90 | 416 |
| ST3GAL3 | 50 | 63 | 90 |
| ST6GALNAC6 | 7 | 11 | 7 |
| ST6GALNAC2 | 1404 | 487 | 1212 |
| ST3GAL4 | 1488 | 2949 | 1814 |
| ST3GAL6 | 143 | 320 | 783 |
| ST6GAL1 | 4342 | 624 | 13710 |
| ST3GAL2 | 1615 | 273 | 2096 |
| ST3GAL1 | 3720 | 2030 | 4470 |
| ST6GALNAC4 | 1492 | 1263 | 1093 |
| ST8SIA1 | 35 | 43 | 230 |
