# Supplementary material for: The role of SIGLEC9 in immunosuppression and prognosis in cervical cancer
Source: Clinics (Sao Paulo). 2025 Dec 18;81:100849. doi: 10.1016/j.clinsp.2025.100849 (PMC12771336; doi:10.1016/j.clinsp.2025.100849)
Supplement: Supplementary file 4 [file mmc4.docx]

ORCID iD

Bihui Wang: 0000-0002-1282-2875

Yuejie Zhu: none

Zhenyu Ru: none

Yulian Zhang: 0009-0009-7988-4032

Mingkai Yu: 0000-0001-6046-2846

Pingfen Li:none

Manli Zhang: 0009-0001-8964-2135

Jianbing Ding: 0000-0001-5506-7665

Zhifang Chen: [0009-0003-1621-5903](https://orcid.org/0009-0003-1621-5903)
